# Supplementary material for: Imaging pain relief in osteoarthritis (IPRO): protocol of a double-blind randomised controlled mechanistic study assessing pain relief and prediction of duloxetine treatment outcome
Source: BMJ Open. 2017 Jun 26;7(6):e014013. doi: 10.1136/bmjopen-2016-014013 (PMC5541453; doi:10.1136/bmjopen-2016-014013)
Supplement: Author's manuscript [file bmjopen-2016-014013.draft_revisions.pdf]

BMJ Open

Imaging Pain Relief in Osteoarthritis (IPRO): protocol of a double blind randomised controlled mechanistic study assessing pain relief and prediction of duloxetine treatment outcome

Journal:	*BMJ Open*
Manuscript ID	bmjopen-2016-014013
Article Type:	Protocol
Date Submitted by the Author:	23-Aug-2016
Complete List of Authors:	Reckziegel, Diane; University of Nottingham, Arthritis Research UK Pain Centre; University of Nottingham, Sir Peter Mansfield Imaging Centre<br>Bailey, Helen; University of Nottingham, Arthritis Research UK Pain Centre; University of Nottingham, Sir Peter Mansfield Imaging Centre<br>Cottam, William; University of Nottingham, Arthritis Research UK Pain Centre; University of Nottingham, Sir Peter Mansfield Imaging Centre<br>Tench, Christopher; University of Nottingham, Division of Clinical Neuroscience<br>Mahajan, Ravi; University of Nottingham, Anaesthesia and Intensive Care<br>Walsh, David; University of Nottingham, Academic Rheumatology<br>Knaggs, Roger; University of Nottingham, School of Pharmacy<br>Auer, Dorothee; University of Nottingham, Arthritis Research UK Pain Centre; University of Nottingham, Sir Peter Mansfield Imaging Centre
**Primary Subject Heading**:	Radiology and imaging
Secondary Subject Heading:	Pharmacology and therapeutics
Keywords:	osteoarthritis pain, analgesia, duloxetine, antidepressant, functional magnetic resonance imaging, MRI

SCHOLARONE™ Manuscripts

For peer review only - http://bmjopen.bmj.com/site/about/guidelines.xhtml

Study protocol

Corresponding author:

Dorothee P Auer, Radiological Sciences, Room W/B 1441, Queen's Medical Centre, Nottingham, NG72UH, UK.

Email: Dorothee.Auer@nottingham.ac.uk

Imaging Pain Relief in Osteoarthritis (IPRO): protocol of a double blind randomised controlled mechanistic study assessing pain relief and prediction of duloxetine treatment outcome

Diane Reckziegel[a,b,c], Helen Bailey[a,b,c], William J Cottam[a,b,c], Christopher R Tench[d], Ravi P Mahajan[a,e], David A Walsh[a,f], Roger D Knaggs[a,g], Dorothee P Auer[a,b,c]

[a]*Arthritis Research UK Pain Centre, University of Nottingham, UK.* [b]*Sir Peter Mansfield Imaging Centre, University of Nottingham, UK.* [c]*Division of Clinical Neuroscience, Radiological Sciences, University of Nottingham, UK.* [d]*Division of Clinical Neuroscience, Clinical Neurology, University of Nottingham, UK.* [e]*Division of Clinical Neuroscience, Anaesthesia and Critical Care, University of Nottingham, UK .*[f]*Division of Rheumatology, Orthopaedics and Dermatology, University of Nottingham, UK.* [g]*School of Pharmacy, University of Nottingham, UK*

We acknowledge other researchers, who significantly contribute to recruitment (Nadia Frowd), help with data collection (Marianne Drabek) and contributed to the initial local internal application (Naranjargal Dashdorj). This work was supported by Arthritis Research UK (Grant 18769) and the University of Nottingham.

For peer review only

Abstract

Introduction: Osteoarthritis (OA) pain is a major cause of long-term disability and chronic pain in the adult population. One in five patients does not receive satisfactory pain relief, which reflects the complexity of chronic pain and the current lack of understanding of mechanisms of chronic pain. Recently, duloxetine has demonstrated clinically relevant pain relief, but only in half of treated patients with OA. Here the aim is to investigate the neural mechanisms of pain relief and neural signatures that may predict treatment response to duloxetine in chronic knee OA pain.

Methods and analysis: This is an ongoing single-centre randomised placebo-controlled mechanistic study (2:1 [placebo] allocation), using a multi-modal neuroimaging approach, together with psychophysiological [quantitative sensory testing], genetics and questionnaire assessments. Eighty-one subjects with chronic knee OA pain are planned to power for between group comparisons (placebo, duloxetine-responder, and duloxetine-non-responder). Participants have a baseline assessment and, following six weeks of duloxetine (30mg for two weeks, then 60mg for four weeks), a follow-up evaluation. Neuroimaging is performed at 3T with blood-oxygen-level dependent functional magnetic resonance imaging at rest and during pin-prick nociceptive stimulation for main outcome assessment; arterial spin labelling and structural imaging (T1-weighted) for secondary outcome assessment. Questionnaires evaluate pain, negative affect, quality of sleep and cognition.

Ethics and dissemination: The study has been approved by the East Midlands – Nottingham 2 research ethics committee (18/EM/0189) and is being carried out under the principles of the Declaration of Helsinki (64th, 2013) and Good Clinical Practice standards. Results will be disseminated in peer-reviewed journals and at scientific conferences.

Trial registration number: This trial is registered at ClinicalTrials.gov (NCT02208778).

This work was supported by Arthritis Research UK (Grant 18769).

Keywords: Osteoarthritis pain, magnetic resonance imaging, fMRI, analgesia, duloxetine, antidepressant

For peer review only

Strengths and limitations of the study

- This study attempts to shed light on the little understood mechanisms of pain relief in chronic pain.
- By undertaking an innovative and integrative approach which combines multi-modal neuroimaging, quantitative sensory testing and psychometric questionnaire assessments, drug effects and predictors of treatment response to duloxetine are aimed to be identified.
- A cross-over design would have ideally been employed. However, a parallel design was chosen in order to minimise dropout rates, which consists of a limitation of the study.

Background and rationale

Osteoarthritis (OA) is a major cause of long-term disability and chronic pain in the adult population. Current treatment options consist of a combination of pharmacological and non-pharmacological alternatives.[1] Non-pharmacological possibilities range from moderate exercise, physiotherapy and cognitive behavioural therapy (CBT) to joint replacement surgery. Pharmacological options include non-steroidal anti-inflammatory drugs (NSAID), opioid analgesics, antidepressants or intra-articular injection of corticosteroids with local anaesthetics.

Despite these options, around 20% of patients do not get satisfactory pain relief, even after joint replacement surgery.[2] In addition, there is currently no protocol to predict efficacy for a given patient, which leads to a trial-and-error approach to treatment which is frustrating for patients and healthcare professionals alike. This is a reflection of the complexity of chronic pain, and the current limited understanding of its underlying mechanisms and how to target these to achieve pain relief. There is converging evidence for chronic pain, including osteoarthritis pain, to be centrally-mediated.[3-5]

For peer review only

Advances of both human and animal neuroimaging tools over the last two decades have improved understanding of brain function across different pain states by providing more objective markers of pain compared to self-report based instruments. It has also allowed more objective measurement of the effects of different types of treatment, as identified in longitudinal studies.[4, 6-8] One promising use of advanced neuroimaging lies in individual endophenotyping of pain mechanisms in clinical patients and through that, guide personalised therapy.[8-10] Such methodologies include functional,[11] morphometric (white matter integrity and grey matter volume) and chemical measures,[12] which can all be used to identify the effects of analgesics and to classify patients into groups according to phenotype.[13]

Brain function can be characterised using functional magnetic resonance imaging (fMRI) techniques such as blood oxygen level dependent (BOLD) contrast or arterial spin labelling (ASL).[14, 15] While ASL allows absolute quantification of blood flow through magnetic tagging, BOLD detects relative changes in concentration ratios of oxy- and deoxy-hemoglobin with increased BOLD signal response, a widely accepted but indirect measure of stimulus-evoked neural activity.[16] To comprehensively describe the brain's activation state, the simultaneous assessment of so-called brain 'signatures' has become popular. Alterations of functional brain networks can be detected using *functional connectivity* (fc) analysis of fMRI data that quantifies the relative temporal synchrony between remote neurophysiological events, enabling the identification and characterisation of brain networks at rest or during an activation state. Advanced connectivity analysis, *effective connectivity,* estimates the influence that one neural system exerts over another based on the temporal ordering of events. Alternative approaches consist of probing known brain regions/networks by predefining a set of *regions of interest* known to be affected in the disease or to mediate an intervention effect. Lastly, *multivariate pattern analysis (MVPA),* which uses a machine-learning approach, has proven an effective way of identifying unknown distributed patterns of neuronal activity.

For peer review only

Numerous studies used such fMRI techniques to describe transient pain in healthy populations and fewer in chronic pain patients.[17] MVPA is increasingly deployed for refined characterisation of brain signatures in response to noxious stimulation in healthy volunteers, and their modulation by analgesic drugs.[8, 10]

Duloxetine, a serotonin and noradrenaline reuptake inhibitor, has antidepressant and anxiolytic properties and is currently also used to treat chronic pain conditions, including ongoing OA pain. However, the precise mechanisms by which this drug acts to relieve pain are unclear and only around 50% of OA patients have sufficient pain relief after duloxetine intake;[18] currently there is no way to predict responders.[18, 19] Functional neuroimaging may improve our understanding of the central processes through which duloxetine provides pain relief and may help identifying predictors of treatment response in chronic OA pain patients. In healthy participants, duloxetine was found to attenuate activity in affect processing areas (amygdala, thalamus, insula and ACC) while simultaneously increasing functional coupling between the amygdala and the anterior insula, during an emotional face matching task.[20] After duloxetine intake but not placebo, major depression disorder patients presented augmented connectivity in the anterior *default mode network (DMN)*,[21] a core network linked to self-referential brain function with distinct abnormalities in major depression.[22] Importantly, reduced connectivity within the orbitofrontal component of the DMN, especially the subgenual cingulate, at baseline was predictive of clinical antidepressant response.[21]

Mood disorders and pain conditions are often comorbid, as it is the case with chronic OA pain.[23] They also show strong similarities in their neurobiological mechanisms and both are known to critically involve limbic neurocircuits.[24, 25] In fact, previously reported brain activity linked to perceived severity of clinical pain may be partially explained by anxiety.[11] This may be specific to clinical pain rating as experimental pain responses were not modulated by negative mood (depressive symptoms, anxiety or catastrophizing) in fibromyalgia.[26] In neuropathic pain, the analgesic efficacy of duloxetine treatment was also independent of

For peer review only

depression, but was predicted by psychophysical measures of impaired conditioned pain modulation (CPM), but not temporal summation (TS).[27] Increased activity in the right pre-fontal cortex may predict the analgesic effect of duloxetine as found in a small pain fMRI study in 13 depressed patients.[25]

The aim of this study is to investigate the neural mechanisms of pain relief in chronic osteoarthritis pain following treatment with duloxetine, and to establish predictors of treatment response. Both resting state functional connectivity (rsfc) and task-related fMRI are used, along with structural T1-weighted MRI and proton spectroscopy, in combination with quantitative sensory testing (QST) and questionnaire assessments. Markers of likely response to duloxetine treatment will be explored at baseline. This analysis may lead to a clearer understanding of the underlying mechanisms of pain relief in osteoarthritis.

It is hypothesized that the responding and non-responding sub-groups of OA patients differ in their limbic brain signatures and may show differences in negative affect and/or sensory pain phenotypes. Additionally, it is hypothesized that duloxetine induces brain network and activation changes that partially overlap with antidepressant mechanisms, especially by improving top down emotional control.

Primary objectives

To identify a functional change in brain signature indexing the analgesic mechanism of duloxetine.

To identify brain imaging and psychophysical markers that predict the response to duloxetine treatment.

For peer review only

Main hypotheses

- Analgesic response to duloxetine treatment can be predicted using a range of baseline brain imaging markers and QST.
- Analgesic response to duloxetine is mediated by modulation of neural networks underpinning emotional control.
- Duloxetine-induced changes in brain activation differ between responders and non-responders.

Design and Methods

This is a single centre, double-blind, placebo controlled mechanistic study being conducted at the University of Nottingham (United Kingdom).

Inclusion criteria

In order to participate in this study, individuals must have chronic knee pain with radiographically defined OA changes, be able to consent to participation and be aged 35 or older. They must be also free of major medical, neurological and psychiatric comorbidities known to affect neural processing of pain.

Exclusion criteria

Potential candidates meeting any of the criteria listed below are excluded from participation.

General:

- Significant medical conditions;

For peer review only

- Refusal by candidate to GP being informed;

MRI-related:

- Intraocular metallic foreign bodies;
- Intracranial aneurysm clips;
- Cardiac pacemakers and defibrillators;
- Cochlear implants;
- Significant head tremor;
- Potential metal foreign bodies due to previous accidents;
- Breastfeeding or pregnancy, confirmed by pregnancy test;
- Unfitness for the MRI scanner, according to the judgement of medically qualified personnel, either on the research team, or the patient's clinical team (*e.g.* due to back pain, claustrophobia, acute sickness etc). This includes patients with signs of impaired temperature regulation such as an extremely high fever;
- Patients with large tattoos, specifically in the head, neck or shoulder region;
- Metallic agents embedded within the body (*i.e.* Shrapnel, surgical pins);

Duloxetine-related:

- Uncontrolled narrow-angle glaucoma;
- Recent usage of monoamine oxidase inhibitor (MAOI) or Mellaril® (thioridazine);
- Taking St. John's Wort (Hypericum perforatum);
- On fluvoxamine, ciprofloxacin or enoxacin;
- Taking other medicines containing duloxetine;

For peer review only

- Liver disease;
- Currently on antidepressant treatment, including treatment for pain with tricyclic agents such as amitriptyline;
- Taking tramadol;
- Known hypersensitivity, allergy or intolerance to one of duloxetine's components;
- Unwillingness to take caution in relation to use of other centrally active substances such as alcohol and sedative drugs;
- Current treatment with potent inhibitors of CYP1A2 like fluvoxamine;
- History of seizures or any drug lowering the seizure threshold

Randomisation and blinding

Participants are randomized between duloxetine (n=54) and placebo (n=27). The allocation ratio is 2:1; given that around half of the population undergoing active treatment is expected to have satisfactory pain relief, this should result in approximately equal numbers between groups of placebo intakes, responders and non-responders. A block randomization, with random block sizes, is utilized, and was generated by the Clinical Trials Pharmacy (CTP) at Nottingham University Hospitals (NHS Trust) using a pseudo-random algorithm at http://www.randomization.com. The allocation list is kept by the CTP in a password-protected file. Investigators may identify the treatment for each participant via request to the CTP, however, during treatment, this breaking the code procedure may only be done in the event of a medical emergency, when treatment is dependent on knowledge of the actual drug received. In case the treatment code for a participant is broken or other actions, such as recording date or reasons for breaking the blind, discontinuation of trial treatment must be reported to the sponsor (University of Nottingham).

For peer review only - http://bmjopen.bmj.com/site/about/guidelines.xhtml

Participants are assigned, by the investigators, a unique identification number (sequentially numbered) and attend the university twice, for both a baseline and follow-up visits. Treatment consists of six weeks of taking either duloxetine capsules or placebo equivalent, followed by two weeks of dose reduction for those taking duloxetine, in order to minimize drug withdrawal effects. In order to minimize potential side effects, participants take one tablet (duloxetine 30mg) daily during two weeks prior to switching to the target dose of 60mg/day. Both researchers and participants are blinded to the treatment allocation until data acquisition is complete. At the end of the follow-up visit, individual allocation is disclosed to the participant only, by one of the CTP staff members, who are not involved in the any data analysis or research procedures. Data analysists will be blinded to treatment allocation.

Individual participation in the study is for around eight weeks, starting with signing informed consent and ending with the conclusion of the drug reduction phase.

(Insert Figure 1)

Experimental testing

The experimental testing is primarily based on multi-modal neuroimaging and QST, in conjunction with questionnaire assessment, genetics and clinical examination of the knee. This should provide a broad evaluation of the multi-faceted nature of pain, assessing both central and peripheral mechanisms, as well as psychological and cognitive traits, among others, which should allow pinpointing precise phenotyping of patients into distinct groups. Further details about the data being collected are given below and summarised in Figure 1.

MR-scan:

For peer review only

- Arterial Spin Labelling (baseline and follow-up);
- Pinprick BOLD fMRI (baseline and follow-up);
- Resting state (stimulus-free) BOLD fMRI (rsfMRI) (baseline and follow-up);
- T1-weighted (baseline and follow-up);
- MR Spectroscopy (MRS) of the mid anterior cingulate cortex (baseline and follow-up).

QST:

- Conditioned Pain Modulation (baseline and follow-up);
- Temporal Summation of Pain (baseline and follow-up);
- Pressure Pain Thresholds (PPT) of the most affected knee and sternum (baseline and follow-up);

Questionnaires:

- PainDetect (baseline);[28]
- Pain Catastrophizing Scale (PCS) (baseline);[29]
- State-Trait Anxiety Inventory (STAI) (baseline);[30]
- Beck Depression Inventory II (baseline);[31]
- ICOAP (baseline and follow-up);[32]
- The pain sleep questionnaire (baseline);[33]
- Brief Pain Inventory (daily);[34]
- Mini-Cog (baseline);[35]
- Educational Level (baseline);

For peer review only

- Questions on belief (baseline);[36]

Participant general details:

- Age
- Gender
- Pain intensity
- Pain duration
- Analgesia intake (last 24h)
- Caffeine intake (last 24h)
- Average daily caffeine intake
- Average weekly alcohol intake
- Medication

Other:

- 10ml blood samples
- Analgesia diary (daily)

Response definition

Response to duloxetine is defined as a 50% reduction in the severity of ongoing pain from baseline to end of treatment.

For peer review only

Primary outcomes

1. Reduction in nociceptive brain response after duloxetine;
2. Neural network change (resting condition) induced by duloxetine ;
3. Differences in brain response and network change between responders and non-responders;
4. Predicting response to duloxetine from baseline fMRI metrics using univariate and multivariate analyses.

Secondary outcomes

I. Identification of QST and questionnaire parameters that predict response to duloxetine.

II. Correlation between baseline CPM and TS with brain activity and connectivity changes.

III. Group differences in brain activity and structure in pain processing, limbic and modulatory pathways changes following duloxetine treatment in comparison to placebo.

Other outcomes

Exploratory outcomes include, but are not limited to, determination of gene variations that can be linked with duloxetine treatment response and GABA modulation according to changes in pain severity.

For peer review only

Data analysis

Parametric maps will be derived for functional and structural parameters and regional activation associated with the functional BOLD changes, along with functional connectivity maps for regions of interest (ACC, insula, brainstem, thalamus and limbic system) and networks.

Image processing and statistical analyses will be performed using the latest versions of statistical and image analysis software such as SPSS (IBM), FSL (FMRIB),[37] PyMVPA,[38] SPM (Statistical Parametric Mapping version 8, London, UK), REST and GRETNA,[39, 40] and LCmodel for MRS data.[41]

Sample size estimation

The sample size calculation was performed according to the guidelines stated by Lenth and colleagues.[42] Estimates of change in functional activation were deduced from a duloxetine intervention study.[20] Using the Java applets [43] it was estimated that a one-sample, one-tailed, t-test would have 80% power at alpha of <0.005 (to allow for ten multiple independent tests) with approximately 19 participants per group: responders to duloxetine treatment, non-responders, and placebo groups.

Based on previous experience of non-usable imaging data due to patient and / or technical factors in 10% of cases, a sample size of >22 participants per group was necessary. A further allowance for 20% dropout gives 27 participants in each group, resulting in a recruitment target of 81 participants.

For peer review only

Recruitment

Identification of potential participants is by contacting people who have taken part in previous studies within the Arthritis Research UK Pain Centre (ARUKPC). Recruitment through primary care and posters is also being used. Candidates are pre-screened during phone call following expression of interest. Medical notes are verified for eligibility following oral consent. Suitable candidates are then booked in for the first visit when written informed consent is obtained from all individuals taking part in the study.

Concomitant medication

Participants are instructed not to change their regular pain treatment while taking part in the study. Rescue pain medication, taken on an 'as needed basis', is accepted during the trial period and its use is documented in a daily pain diary.

Criteria for withdrawal

Participation in this study is voluntary and participants are free to withdraw from the trial at any time, without giving any reason. However they must seek advice from the researchers before doing so as discontinuing duloxetine treatment suddenly may cause drug withdrawal effects. Involvement may be also discontinued by the investigators in case of any significant adverse effect of duloxetine/placebo treatment, or when subjects require major changes to their ongoing pain management, during the course of the study.

For peer review only

Adverse events and data safety monitoring

All observed or reported adverse events (AEs) are recorded by the investigators. No AEs are expected in relation to the MRI scanning. Potential AEs are anticipated to be only related to drug intake. All adverse events are recorded and monitored until resolution, stabilisation, or until it has been shown that the study intervention is not the cause. Any serious adverse event (SAE) is recorded and reported to the research ethics committee (REC) as part of the annual reports.

Handling and storage of data and documents

Personal data is handled confidentially. Imaging, sample and questionnaire data are anonymised by assigning a unique participant identification code, containing the abbreviation of the study's name followed by a sequence number. The link between study identification code and individual's personal details is kept on the consent form and MR screening form.

Ethics and dissemination

The study was approved by the East Midlands – Nottingham 2 research ethics committee (18/EM/0189) in August 2014 and is being carried out under the principles of the Declaration of Helsinki (64th, 2013) and Good Clinical Practice standards. Any modifications to the original protocol must undergo review by the research ethics committee involved. The design, documentation and data collection have been additionally discussed and shaped according to opinion of a focus group formed by representatives of patients and the public. The first participant was recruited in December 2014 and the last follow-up visit is expected in September 2016. Results will be disseminated in peer-reviewed journals and at scientific meetings and conferences, where patient data will be presented anonymously.

For peer review only

Funding

This work is supported by Arthritis Research UK (Grant 18769).

Contributors

DPA and DAW designed the study and DR, DPA, RDK, CRT and RPM developed the research protocol. DR coordinates the study, is responsible for data acquisition and will conduct image and statistical analyses. CRT provides statistical consultation. DR, HB and WC undertake recruitment and data collection. DR and DPA wrote the manuscript, which was edited by CRT and RDK. All authors approved the final manuscript.

Competing interests

The authors have no potential conflicts of interest with respect to the research, authorship and publication of this article.

References

1. McAlindon TE, Bannuru RR, Sullivan M, et al. OARSI guidelines for the non-surgical management of knee osteoarthritis. *Osteoarthritis and cartilage* 2014;22(3):363-88
2. Beswick AD, Wylde V, Gooberman-Hill R, Blom A, Dieppe P. What proportion of patients report long-term pain after total hip or knee replacement for osteoarthritis? A systematic review of prospective studies in unselected patients. *BMJ open* 2012;2(1):e000435
3. Ohtori S, Orita S, Yamashita M, et al. Existence of a neuropathic pain component in patients with osteoarthritis of the knee. *Yonsei medical journal* 2012;53(4):801-05
4. Sanders D, Krause K, O'Muircheartaigh J, et al. Pharmacologic Modulation of Hand Pain in Osteoarthritis: A Double-Blind Placebo-Controlled Functional Magnetic Resonance Imaging Study Using Naproxen. *Arthritis & Rheumatology* 2015;67(3):741-51

For peer review only

5. Vachon-Presseau E, Tétreault P, Petre B, et al. Corticolimbic anatomical characteristics predetermine risk for chronic pain. *Brain* 2016:aww100
6. Petzke F, Jensen K, Kosek E, et al. Using fMRI to evaluate the effects of milnacipran on central pain processing in patients with fibromyalgia. *Scandinavian Journal of Pain* 2013;4(2):65-74
7. Giménez M, Pujol J, Ali Z, et al. Naproxen effects on brain response to painful pressure stimulation in patients with knee osteoarthritis: a double-blind, randomized, placebo-controlled, single-dose study. *The Journal of rheumatology* 2014;41(11):2240-48
8. Duff EP, Vennart W, Wise RG, et al. Learning to identify CNS drug action and efficacy using multistudy fMRI data. *Science translational medicine* 2015;7(274):274ra16-74ra16
9. Tracey I. Can neuroimaging studies identify pain endophenotypes in humans? *Nature Reviews Neurology* 2011;7(3):173-81
10. Wager TD, Atlas LY, Lindquist MA, Roy M, Woo C-W, Kross E. An fMRI-based neurologic signature of physical pain. *New England Journal of Medicine* 2013;368(15):1388-97
11. Cottam WJ, Condon L, Alshuft H, Reckziegel D, Auer DP. Associations of limbic-affective brain activity and severity of ongoing chronic arthritis pain are explained by trait anxiety. *NeuroImage: Clinical* 2016;12:269-76
12. Reckziegel D, Raschke F, Cottam WJ, Auer DP. Cingulate GABA levels inversely correlate with the intensity of ongoing chronic knee osteoarthritis pain. *Molecular pain* 2016;12:1744806916650690
13. Borsook D, Becerra L, Hargreaves R. Biomarkers for chronic pain and analgesia. Part 2: how, where, and what to look for using functional imaging. *Discovery medicine* 2011;11(58):209-19
14. Brown GG, Perthen JE, Liu TT, Buxton RB. A primer on functional magnetic resonance imaging. *Neuropsychology review* 2007;17(2):107-25
15. Petersen E, Zimine I, Ho YL, Golay X. Non-invasive measurement of perfusion: a critical review of arterial spin labelling techniques. *The British journal of radiology* 2014
16. Ogawa S, Lee T-M, Kay AR, Tank DW. Brain magnetic resonance imaging with contrast dependent on blood oxygenation. *Proceedings of the National Academy of Sciences* 1990;87(24):9868-72
17. Tanasescu R, Cottam WJ, Condon L, Tench CR, Auer DP. Functional reorganisation in chronic pain and neural correlates of pain sensitisation: A coordinate based meta-analysis of 266 cutaneous pain fMRI studies. *Neuroscience & Biobehavioral Reviews* 2016;68:120-33
18. Chappell AS, Ossanna MJ, Liu-Seifert H, et al. Duloxetine, a centrally acting analgesic, in the treatment of patients with osteoarthritis knee pain: a 13-week, randomized, placebo-controlled trial. *Pain* 2009;146(3):253-60
19. Chappell AS, Desaiah D, Liu-Seifert H, et al. A double-blind, randomized, placebo-controlled study of the efficacy and safety of duloxetine for the treatment of chronic pain due to osteoarthritis of the knee. *Pain Practice* 2011;11(1):33-41

For peer review only

20. Van Marle HJ, Tendolkar I, Urner M, Verkes RJ, Fernández G, Van Wingen G. Subchronic duloxetine administration alters the extended amygdala circuitry in healthy individuals. *Neuroimage* 2011;55(2):825-31
21. Fu CH, Costafreda SG, Sankar A, et al. Multimodal functional and structural neuroimaging investigation of major depressive disorder following treatment with duloxetine. *BMC psychiatry* 2015;15(1):1
22. Sheline YI, Barch DM, Price JL, et al. The default mode network and self-referential processes in depression. *Proceedings of the National Academy of Sciences* 2009;106(6):1942-47
23. Bair MJ, Wu J, Damush TM, Sutherland JM, Kroenke K. Association of depression and anxiety alone and in combination with chronic musculoskeletal pain in primary care patients. *Psychosomatic medicine* 2008;70(8):890
24. Baliki MN, Apkarian AV. Nociception, pain, negative moods, and behavior selection. *Neuron* 2015;87(3):474-91
25. López-Solà M, Pujol J, Hernández-Ribas R, et al. Effects of duloxetine treatment on brain response to painful stimulation in major depressive disorder. *Neuropsychopharmacology* 2010;35(11):2305-17
26. Jensen KB, Petzke F, Carville S, et al. Anxiety and depressive symptoms in fibromyalgia are related to poor perception of health but not to pain sensitivity or cerebral processing of pain. *Arthritis & Rheumatism* 2010;62(11):3488-95
27. Yarnitsky D, Granot M, Nahman-Averbuch H, Khamaisi M, Granovsky Y. Conditioned pain modulation predicts duloxetine efficacy in painful diabetic neuropathy. *Pain* 2012;153(6):1193-98
28. Freynhagen R, Baron R, Gockel U, Tölle TR. Pain DETECT: a new screening questionnaire to identify neuropathic components in patients with back pain. *Current medical research and opinion* 2006;22(10):1911-20
29. Sullivan MJ, Bishop SR, Pivik J. The pain catastrophizing scale: development and validation. *Psychological assessment* 1995;7(4):524
30. Spielberger CD. *State-Trait anxiety inventory*: Wiley Online Library, 2010.
31. Beck AT, Steer RA, Brown GK. Beck depression inventory-II. *San Antonio, TX* 1996:78204-2498
32. Hawker G, Davis A, French M, et al. Development and preliminary psychometric testing of a new OA pain measure–an OARSI/OMERACT initiative. *Osteoarthritis and Cartilage* 2008;16(4):409-14
33. Ayearst LE, Harsanyi Z, Michalko KJ. The Pain and Sleep Questionnaire three-item index (PSQ-3): A reliable and valid measure of the impact of pain on sleep in chronic nonmalignant pain of various etiologies. *Pain Research and Management* 2012;17(4):281-90
34. Cleeland C, Ryan K. Pain assessment: global use of the Brief Pain Inventory. *Annals of the Academy of Medicine, Singapore* 1994;23(2):129-38

For peer review only

35. Borson S, Scanlan J, Brush M, Vitaliano P, Dokmak A. The Mini-Cog: a cognitive'vital signs' measure for dementia screening in multi-lingual elderly. *International journal of geriatric psychiatry* 2000;15(11):1021-27
36. Pouli N, Nair RD, Lincoln NB, Walsh D. The experience of living with knee osteoarthritis: exploring illness and treatment beliefs through thematic analysis. *Disability and rehabilitation* 2014
37. Jenkinson M, Beckmann CF, Behrens TE, Woolrich MW, Smith SM. Fsl. *Neuroimage* 2012;62(2):782-90
38. Hanke M, Halchenko YO, Sederberg PB, et al. PyMVPA: a unifying approach to the analysis of neuroscientific data. *Python in Neuroscience* 2015:157
39. Song X-W, Dong Z-Y, Long X-Y, et al. REST: a toolkit for resting-state functional magnetic resonance imaging data processing. *PloS one* 2011;6(9):e25031
40. Wang J, Wang X, Xia M, Liao X, Evans A, He Y. GRETNA: a graph theoretical network analysis toolbox for imaging connectomics. *Frontiers in human neuroscience* 2015;9
41. Provencher SW. Estimation of metabolite concentrations from localized in vivo proton NMR spectra. *Magnetic resonance in medicine* 1993;30(6):672-79
42. Lenth RV. Some practical guidelines for effective sample size determination. *The American Statistician* 2001;55(3):187-93
43. Lenth RV. Java applets for power and sample size [computer software]. *Retrieved October* 2006;31:2013

For peer review only

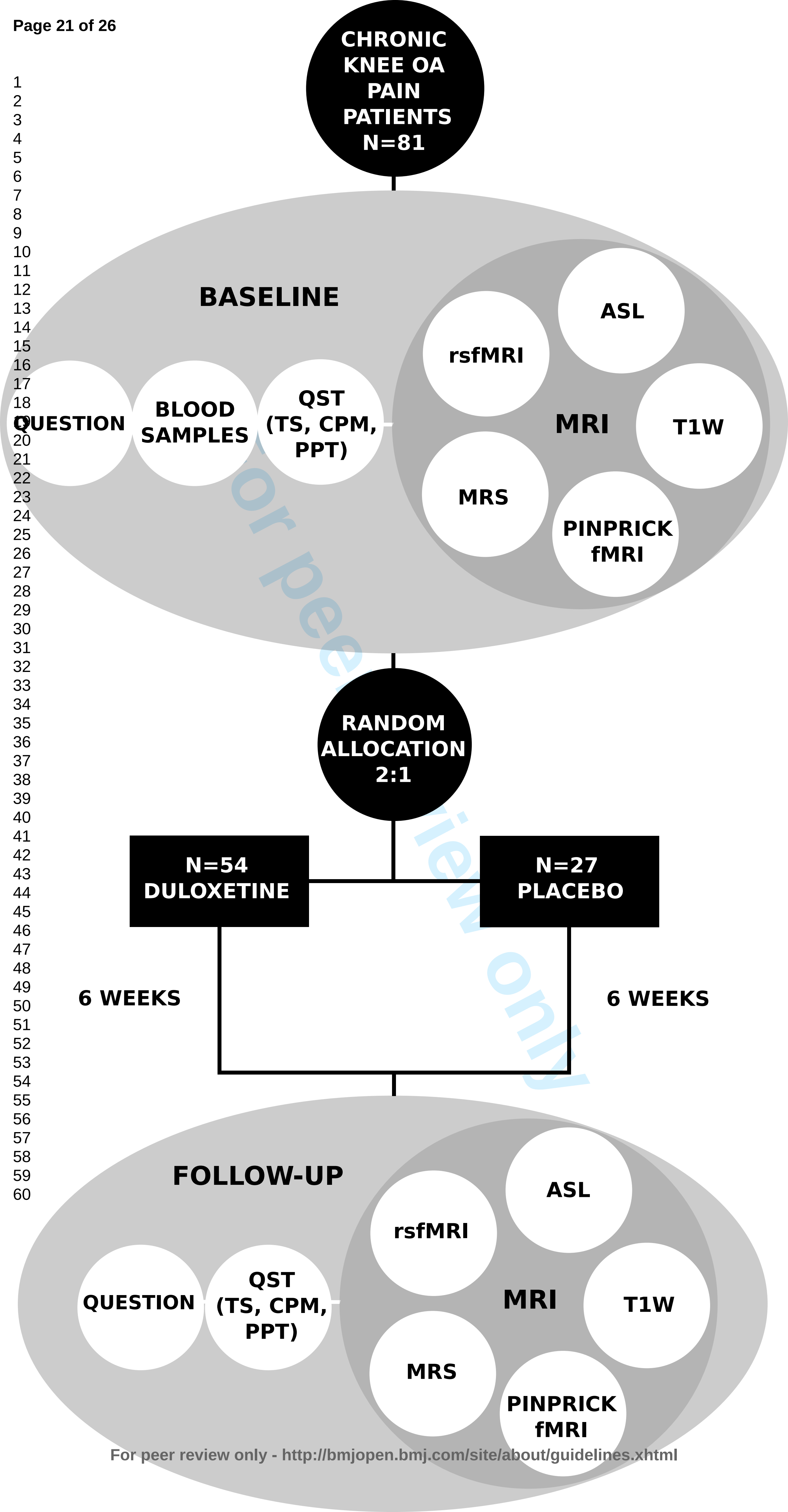

For peer review only - http://bmjopen.bmj.com/site/about/guidelines.xhtml

SPIRIT

STANDARD PROTOCOL ITEMS: RECOMMENDATIONS FOR INTERVENTIONAL TRIALS

SPIRIT 2013 Checklist: Recommended items to address in a clinical trial protocol and related documents*

Section/item	Item No	Description	Addressed on page number
**Administrative information**			
Title	1	Descriptive title identifying the study design, population, interventions, and, if applicable, trial acronym	______1______
Trial registration	2a	Trial identifier and registry name. If not yet registered, name of intended registry	______2______
	2b	All items from the World Health Organization Trial Registration Data Set	______ok_____
Protocol version	3	Date and version identifier	______NA______
Funding	4	Sources and types of financial, material, and other support	______2______
Roles and responsibilities	5a	Names, affiliations, and roles of protocol contributors	______1,16___
	5b	Name and contact information for the trial sponsor	_____10______
	5c	Role of study sponsor and funders, if any, in study design; collection, management, analysis, and interpretation of data; writing of the report; and the decision to submit the report for publication, including whether they will have ultimate authority over any of these activities	_____10_______
	5d	Composition, roles, and responsibilities of the coordinating centre, steering committee, endpoint adjudication committee, data management team, and other individuals or groups overseeing the trial, if applicable (see Item 21a for data monitoring committee)	_____ok______

For peer review only - http://bmjopen.bmj.com/site/about/guidelines.xhtml

For peer review only

**Introduction**			
Background and rationale	6a	Description of research question and justification for undertaking the trial, including summary of relevant studies (published and unpublished) examining benefits and harms for each intervention	____3,4,5,6_____
	6b	Explanation for choice of comparators	____________
Objectives	7	Specific objectives or hypotheses	___5,6,7______
Trial design	8	Description of trial design including type of trial (eg, parallel group, crossover, factorial, single group), allocation ratio, and framework (eg, superiority, equivalence, noninferiority, exploratory)	___7,10_______
**Methods: Participants, interventions, and outcomes**			
Study setting	9	Description of study settings (eg, community clinic, academic hospital) and list of countries where data will be collected. Reference to where list of study sites can be obtained	_____7_______
Eligibility criteria	10	Inclusion and exclusion criteria for participants. If applicable, eligibility criteria for study centres and individuals who will perform the interventions (eg, surgeons, psychotherapists)	_____7,8,9_____
Interventions	11a	Interventions for each group with sufficient detail to allow replication, including how and when they will be administered	_____10______
	11b	Criteria for discontinuing or modifying allocated interventions for a given trial participant (eg, drug dose change in response to harms, participant request, or improving/worsening disease)	_____15______
	11c	Strategies to improve adherence to intervention protocols, and any procedures for monitoring adherence (eg, drug tablet return, laboratory tests)	______________
	11d	Relevant concomitant care and interventions that are permitted or prohibited during the trial	_____14______
Outcomes	12	Primary, secondary, and other outcomes, including the specific measurement variable (eg, systolic blood pressure), analysis metric (eg, change from baseline, final value, time to event), method of aggregation (eg, median, proportion), and time point for each outcome. Explanation of the clinical relevance of chosen efficacy and harm outcomes is strongly recommended	____13,14___
Participant timeline	13	Time schedule of enrolment, interventions (including any run-ins and washouts), assessments, and visits for participants. A schematic diagram is highly recommended (see Figure)	____10,11____

For peer review only - http://bmjopen.bmj.com/site/about/guidelines.xhtml

Sample size	14	Estimated number of participants needed to achieve study objectives and how it was determined, including clinical and statistical assumptions supporting any sample size calculations	______9______
Recruitment	15	Strategies for achieving adequate participant enrolment to reach target sample size	______14_____
**Methods: Assignment of interventions (for controlled trials)**			
Allocation:			
Sequence generation	16a	Method of generating the allocation sequence (eg, computer-generated random numbers), and list of any factors for stratification. To reduce predictability of a random sequence, details of any planned restriction (eg, blocking) should be provided in a separate document that is unavailable to those who enrol participants or assign interventions	______10_____
Allocation concealment mechanism	16b	Mechanism of implementing the allocation sequence (eg, central telephone; sequentially numbered, opaque, sealed envelopes), describing any steps to conceal the sequence until interventions are assigned	_____10______
Implementation	16c	Who will generate the allocation sequence, who will enrol participants, and who will assign participants to interventions	_____10______
Blinding (masking)	17a	Who will be blinded after assignment to interventions (eg, trial participants, care providers, outcome assessors, data analysts), and how	_____10______
	17b	If blinded, circumstances under which unblinding is permissible, and procedure for revealing a participant's allocated intervention during the trial	_____10______
**Methods: Data collection, management, and analysis**			
Data collection methods	18a	Plans for assessment and collection of outcome, baseline, and other trial data, including any related processes to promote data quality (eg, duplicate measurements, training of assessors) and a description of study instruments (eg, questionnaires, laboratory tests) along with their reliability and validity, if known. Reference to where data collection forms can be found, if not in the protocol	____10,11,12___
	18b	Plans to promote participant retention and complete follow-up, including list of any outcome data to be collected for participants who discontinue or deviate from intervention protocols	_____________

For peer review only - http://bmjopen.bmj.com/site/about/guidelines.xhtml

Data management	19	Plans for data entry, coding, security, and storage, including any related processes to promote data quality (eg, double data entry; range checks for data values). Reference to where details of data management procedures can be found, if not in the protocol	______________
Statistical methods	20a	Statistical methods for analysing primary and secondary outcomes. Reference to where other details of the statistical analysis plan can be found, if not in the protocol	_____14______
	20b	Methods for any additional analyses (eg, subgroup and adjusted analyses)	____13,14______
	20c	Definition of analysis population relating to protocol non-adherence (eg, as randomised analysis), and any statistical methods to handle missing data (eg, multiple imputation)	______________
**Methods: Monitoring**			
Data monitoring	21a	Composition of data monitoring committee (DMC); summary of its role and reporting structure; statement of whether it is independent from the sponsor and competing interests; and reference to where further details about its charter can be found, if not in the protocol. Alternatively, an explanation of why a DMC is not needed	_____NA_______
	21b	Description of any interim analyses and stopping guidelines, including who will have access to these interim results and make the final decision to terminate the trial	_____NA______
Harms	22	Plans for collecting, assessing, reporting, and managing solicited and spontaneously reported adverse events and other unintended effects of trial interventions or trial conduct	_____15,16_____
Auditing	23	Frequency and procedures for auditing trial conduct, if any, and whether the process will be independent from investigators and the sponsor	______________
**Ethics and dissemination**			
Research ethics approval	24	Plans for seeking research ethics committee/institutional review board (REC/IRB) approval	_____2, 16____
Protocol amendments	25	Plans for communicating important protocol modifications (eg, changes to eligibility criteria, outcomes, analyses) to relevant parties (eg, investigators, REC/IRBs, trial participants, trial registries, journals, regulators)	_____16______

For peer review only - http://bmjopen.bmj.com/site/about/guidelines.xhtml

Consent or assent	26a	Who will obtain informed consent or assent from potential trial participants or authorised surrogates, and how (see Item 32)	____14_______
	26b	Additional consent provisions for collection and use of participant data and biological specimens in ancillary studies, if applicable	____14________
Confidentiality	27	How personal information about potential and enrolled participants will be collected, shared, and maintained in order to protect confidentiality before, during, and after the trial	____16_______
Declaration of interests	28	Financial and other competing interests for principal investigators for the overall trial and each study site	____16,17_____
Access to data	29	Statement of who will have access to the final trial dataset, and disclosure of contractual agreements that limit such access for investigators	____16_______
Ancillary and post-trial care	30	Provisions, if any, for ancillary and post-trial care, and for compensation to those who suffer harm from trial participation	____ok________
Dissemination policy	31a	Plans for investigators and sponsor to communicate trial results to participants, healthcare professionals, the public, and other relevant groups (eg, via publication, reporting in results databases, or other data sharing arrangements), including any publication restrictions	_____________
	31b	Authorship eligibility guidelines and any intended use of professional writers	____16_______
	31c	Plans, if any, for granting public access to the full protocol, participant-level dataset, and statistical code	____ok_______
**Appendices**			
Informed consent materials	32	Model consent form and other related documentation given to participants and authorised surrogates	_____ok_______
Biological specimens	33	Plans for collection, laboratory evaluation, and storage of biological specimens for genetic or molecular analysis in the current trial and for future use in ancillary studies, if applicable	____ok_______

*It is strongly recommended that this checklist be read in conjunction with the SPIRIT 2013 Explanation & Elaboration for important clarification on the items. Amendments to the protocol should be tracked and dated. The SPIRIT checklist is copyrighted by the SPIRIT Group under the Creative Commons "Attribution-NonCommercial-NoDerivs 3.0 Unported" license.

For peer review only - http://bmjopen.bmj.com/site/about/guidelines.xhtml

BMJ Open

Imaging Pain Relief in Osteoarthritis (IPRO): protocol of a double blind randomised controlled mechanistic study assessing pain relief and prediction of duloxetine treatment outcome

Journal:	*BMJ Open*
Manuscript ID	bmjopen-2016-014013.R1
Article Type:	Protocol
Date Submitted by the Author:	27-Dec-2016

Complete List of Authors:	Reckziegel, Diane; University of Nottingham, Arthritis Research UK Pain Centre; University of Nottingham, Sir Peter Mansfield Imaging Centre<br>Bailey, Helen; University of Nottingham, Arthritis Research UK Pain Centre; University of Nottingham, Sir Peter Mansfield Imaging Centre<br>Cottam, William; University of Nottingham, Arthritis Research UK Pain Centre; University of Nottingham, Sir Peter Mansfield Imaging Centre<br>Tench, Christopher; University of Nottingham, Division of Clinical Neuroscience<br>Mahajan, Ravi; University of Nottingham, Anaesthesia and Intensive Care<br>Walsh, David; University of Nottingham, Academic Rheumatology<br>Knaggs, Roger; University of Nottingham, School of Pharmacy<br>Auer, Dorothee; University of Nottingham, Arthritis Research UK Pain Centre; University of Nottingham, Sir Peter Mansfield Imaging Centre

**Primary Subject Heading**:	Radiology and imaging
Secondary Subject Heading:	Pharmacology and therapeutics
Keywords:	osteoarthritis pain, analgesia, duloxetine, antidepressant, functional magnetic resonance imaging, MRI

SCHOLARONE™
Manuscripts

Study protocol

Corresponding author:

Dorothee P Auer, Radiological Sciences, Room W/B 1441, Queen's Medical Centre, Nottingham, NG72UH, UK.

Email: Dorothee.Auer@nottingham.ac.uk

Imaging Pain Relief in Osteoarthritis (IPRO): protocol of a double blind randomised controlled mechanistic study assessing pain relief and prediction of duloxetine treatment outcome

Diane Reckziegel[a,b,c], Helen Bailey[a,b,c], William J Cottam[a,b,c], Christopher R Tench[d], Ravi P Mahajan[a,e], David A Walsh[a,f], Roger D Knaggs[a,g], Dorothee P Auer[a,b,c]

[a]*Arthritis Research UK Pain Centre, University of Nottingham, UK.* [b]*Sir Peter Mansfield Imaging Centre, University of Nottingham, UK.* [c]*Division of Clinical Neuroscience, Radiological Sciences, University of Nottingham, UK.* [d]*Division of Clinical Neuroscience, Clinical Neurology, University of Nottingham, UK.* [e]*Division of Clinical Neuroscience, Anaesthesia and Critical Care, University of Nottingham, UK .*[f]*Division of Rheumatology, Orthopaedics and Dermatology, University of Nottingham, UK.* [g]*School of Pharmacy, University of Nottingham, UK*

We acknowledge other researchers, who significantly contribute to recruitment (Nadia Frowd), help with data collection (Marianne Drabek) and contributed to the initial local internal application (Naranjargal Dashdorj). This work was supported by Arthritis Research UK (Grant 18769) and the University of Nottingham.

Abstract

Introduction: Osteoarthritis (OA) pain is a major cause of long-term disability and chronic pain in the adult population. One in five patients does not receive satisfactory pain relief, which reflects the complexity of chronic pain and the current lack of understanding of mechanisms of chronic pain. Recently, duloxetine has demonstrated clinically relevant pain relief, but only in half of treated patients with OA. Here the aim is to investigate the neural mechanisms of pain relief and neural signatures that may predict treatment response to duloxetine in chronic knee OA pain.

Methods and analysis: This is an ongoing single-centre randomised placebo-controlled mechanistic study (2:1 [placebo] allocation), using a multi-modal neuroimaging approach, together with psychophysiological [quantitative sensory testing], genetics and questionnaire assessments. Eighty-one subjects with chronic knee OA pain are planned to power for between group comparisons (placebo, duloxetine-responder, and duloxetine-non-responder). Participants have a baseline assessment and, following six weeks of duloxetine (30mg for two weeks, then 60mg for four weeks), a follow-up evaluation. Brain imaging is performed at 3T with blood-oxygen-level dependent functional magnetic resonance imaging at rest and during pin-prick nociceptive stimulation for main outcome assessment; arterial spin labelling and structural imaging (T1-weighted) for secondary outcome assessment. Questionnaires evaluate pain, negative affect, quality of sleep and cognition.

Ethics and dissemination: The study has been approved by the East Midlands – Nottingham 2 research ethics committee (18/EM/0189) and is being carried out under the principles of the Declaration of Helsinki (64th, 2013) and Good Clinical Practice standards. Results will be disseminated in peer-reviewed journals and at scientific conferences.

Trial registration number: This trial is registered at ClinicalTrials.gov (NCT02208778).

This work was supported by Arthritis Research UK (Grant 18769).

Keywords: Osteoarthritis pain, magnetic resonance imaging, fMRI, analgesia, duloxetine, antidepressant

For peer review only

Strengths and limitations of the study

- This study attempts to shed light on the little understood mechanisms of pain relief in chronic pain.
- By undertaking an innovative and integrative approach which combines multi-modal neuroimaging, quantitative sensory testing and psychometric questionnaire assessments, drug effects and predictors of treatment response to duloxetine are aimed to be identified.
- A cross-over design would have ideally been employed. However, a parallel design was chosen in order to minimise dropout rates.

Background and rationale

Osteoarthritis (OA) is a major cause of long-term disability and chronic pain in the adult population. Current treatment options consist of a combination of pharmacological and non-pharmacological alternatives.[1] Despite these options, around 20% of patients do not get satisfactory pain relief, even after joint replacement surgery.[2] This strongly supports the notion that chronic osteoarthritis pain has a strong central component[3-5]

Application of advanced neuroimaging tools led to novel understanding of central mechanisms underpinning acute and chronic pain states.[6] Functional imaging in particular lends itself to the mechanistic study of analgesic treatment.[4 7-9] Neuroimaging may also prove useful in guiding mechanism-based personalised therapy.[9-11]

To comprehensively describe the brain's activation state across regions and functional properties, the concept of so-called brain 'signatures' has emerged that can be derived from regional and network analysis of blood oxygen level dependent (BOLD) functional MRI data at

For peer review only

rest or during controlled stimulation, or from cerebral blood flow maps based on arterial spin labelling (ASL) techniques.[12 13]

Duloxetine, a serotonin and noradrenaline reuptake inhibitor, has antidepressant and anxiolytic properties and is currently also used to treat chronic pain conditions. However, the precise mechanisms by which this drug acts to relieve pain are unclear and only around 50% of OA patients have sufficient pain relief after duloxetine intake;[14] currently there is no way to predict responders.[14 15] In healthy participants, duloxetine was found to attenuate activity in affect processing areas (amygdala, thalamus, insula and ACC) while simultaneously increasing functional coupling between the amygdala and the anterior insula, during an emotional face matching task.[16] Duloxetine in major depression augmented connectivity in the anterior *default mode network (DMN)*,[17] while reduced connectivity within the subgenual cingulate was predictive of clinical antidepressant response.[17]

The aim of this study is to investigate the neural mechanisms of pain relief in chronic osteoarthritis pain following treatment with duloxetine, and to establish multimodal brain signatures to predict treatment response. We will use a unique multimodal MRI design to identify key mechanisms and predictors of duloxetine-induced pain relief in chronic OA pain. This will include brain resting state functional connectivity (rsfc), ASL, nociceptive fMRI, structural T1-weighted brain MRI, proton spectroscopy, quantitative sensory testing (QST) and questionnaire assessments.

We hypothesize that responding and non-responding OA patients have different limbic signatures, differences in negative affect and sensory pain phenotypes. Additionally, we hypothesize that duloxetine induces functional brain changes in emotional regulatory networks.

For peer review only

Primary objectives

To identify a functional change in brain signature indexing the analgesic mechanism of duloxetine.

To identify brain imaging and psychophysical markers that predict the response to duloxetine treatment.

Main hypotheses

- Analgesic response to duloxetine treatment can be predicted using a range of baseline brain imaging markers and QST.
- Analgesic response to duloxetine is mediated by modulation of neural networks underpinning emotional control.
- Duloxetine-induced changes in brain activation differ between responders and non-responders.

Design and Methods

This is a single centre, double-blind, placebo controlled mechanistic study being conducted at the University of Nottingham (United Kingdom).

Inclusion criteria

In order to participate in this study, individuals must have chronic knee pain with radiographically defined OA changes (KLG 2 or higher), be able to consent to participation and

For peer review only

be aged 35 or older. They must also be free of major medical, neurological and psychiatric comorbidities known to affect neural processing of pain.

Exclusion criteria

Potential candidates meeting any of the criteria listed below are excluded from participation.

General:

- Significant medical conditions, including uncontrolled hypertension, insulin dependent diabetes, dementia, Parkinson's disease, epilepsy, multiple sclerosis, active cancer, major depressive disorder, mania, bipolar and schizophrenia. This is judged by a qualified physician based on medical records;
- Refusal by candidate to GP being informed;
- Planned total knee replacement;

MRI-related:

- Intraocular metallic foreign bodies;
- Intracranial aneurysm clips;
- Cardiac pacemakers and defibrillators;
- Cochlear implants;
- Significant head tremor;
- Potential metal foreign bodies due to previous accidents;
- Breastfeeding or pregnancy, confirmed by pregnancy test;

For peer review only

- Unfitness for the MRI scanner, according to the judgement of medically qualified personnel, either on the research team, or the patient's clinical team (*e.g.* due to back pain, claustrophobia, acute sickness etc). This includes patients with signs of impaired temperature regulation such as an extremely high fever;
- Patients with large tattoos, specifically in the head, neck or shoulder region;
- Metallic agents embedded within the body (*i.e.* Shrapnel, surgical pins);

Duloxetine-related:

- Uncontrolled narrow-angle glaucoma;
- Recent usage of monoamine oxidase inhibitor (MAOI) or Mellaril® (thioridazine);
- Taking St. John's Wort (Hypericum perforatum);
- On fluvoxamine, ciprofloxacin or enoxacin;
- Taking other medicines containing duloxetine;
- Liver disease;
- Renal impairment;
- Currently on antidepressant treatment, including treatment for pain with tricyclic agents such as amitriptyline;
- Taking tramadol;
- Known hypersensitivity, allergy or intolerance to one of duloxetine's components;
- Unwillingness to take caution in relation to use of other centrally active substances such as alcohol and sedative drugs;
- Current treatment with potent inhibitors of CYP1A2 like fluvoxamine;

For peer review only

- History of seizures or any drug lowering the seizure threshold

Randomisation and blinding

Participants are randomized between duloxetine (n=54) and placebo (n=27). The allocation ratio is 2:1; given that around half of the population undergoing active treatment is expected to have satisfactory pain relief, this should result in approximately equal numbers between groups of placebo intakes, responders and non-responders. A block randomization, with block sizes of 9 and 2, is utilized, and was generated by the Clinical Trials Pharmacy (CTP) at Nottingham University Hospitals (NHS Trust) using a pseudo-random algorithm at http://www.randomization.com. The allocation list is kept by the CTP in a password-protected file. Investigators may identify the treatment for each participant via request to the CTP, however, during treatment, this breaking the code procedure may only be done in the event of a medical emergency, when treatment is dependent on knowledge of the actual drug received. In case the treatment code for a participant is broken or other actions, such as recording date or reasons for breaking the blind, discontinuation of trial treatment must be reported to the sponsor (University of Nottingham).

(Insert Figure 1)

Participants are assigned, by the investigators, a unique identification number (sequentially numbered) and attend the university twice, for both a baseline and follow-up visits. Treatment consists of six weeks of taking either duloxetine capsules or placebo equivalent, followed by two weeks of dose reduction for those taking duloxetine, in order to minimize drug withdrawal effects (Figure 1). In order to minimize potential side effects, participants take one tablet (duloxetine 30mg) daily during two weeks prior to switching to the target dose of 60mg/day.

Both researchers and participants are blinded to the treatment allocation until data acquisition is complete. At the end of the follow-up visit, individual allocation is disclosed to the participant only, by one of the CTP staff members, who are not involved in the any data analysis or research procedures. Data analysts will be blinded to treatment allocation.

Individual participation in the study is for around eight weeks, starting with signing informed consent and ending with the conclusion of the drug reduction phase.

(Insert Figure 2)

Experimental testing

The experimental testing is primarily based on multi-modal neuroimaging and QST, in conjunction with questionnaire assessment and genetics. This should provide a broad evaluation of the multi-faceted nature of pain, assessing both central and peripheral mechanisms, as well as psychological and cognitive traits, among others, which should allow pinpointing precise phenotyping of patients into distinct groups. Time points and types of data being collected are summarised in Figure 2 and details of questionnaires are given below.

Questionnaires:

- PainDetect (baseline) – a screening questionnaire to identify neuropathic components of pain;[18]
- Pain Catastrophizing Scale (PCS) (baseline) – a 13-item scale to measure pain catastrophizing, which can be split into three sub-scales named rumination, magnification and helplessness;[19]

For peer review only

- State-Trait Anxiety Inventory (STAI) (baseline) – a psychological inventory assessing anxiety. It contains 40 items and can be split into two sub-scales being state anxiety and trait anxiety;[20]
- Beck Depression Inventory II (BDI) (baseline) – a 21-item multiple-choice self-report scale for measuring the severity of depression;[21]
- The intermittent and constant pain score (ICOAP) (baseline and follow-up) – an 11-question scale to characterise pain in individuals with osteoarthritis pain. It gives two sub-scales relating to intermittent and constant experience of pain;[22]
- The pain sleep questionnaire (baseline) – a 5-item tool to assess the impact of pain on sleep;[23]
- Brief Pain Inventory (BPI) (daily) – a 9-item questionnaire for measuring the severity of pain and the impact of this pain on daily functioning. The severity sub-scale is used to define the response definition (below);[24]
- Mini-Cog (baseline) – contains a 3-item recall test and a clock drawing test to screen cognitive impairment;[25]
- Educational Level (baseline);
- Questions on belief (baseline) – consist of four questions to assess an individual's expectations on treatment and medical care;[26]

Participant general details:

- Age;
- Sex;

For peer review only

- Pain intensity (measured on a numeric rating scale ranging from 0-100, where 0 represents no pain at all and 100 stands for excruciating pain);Pain duration;
- Analgesia intake (last 24h);
- Caffeine intake (last 24h);
- Average daily caffeine intake;
- Average weekly alcohol intake;
- Medication;

Other:

- 10ml blood samples;
- Analgesia diary (daily).

Response definition

Response to duloxetine is defined as a 50% reduction in the severity of ongoing pain from baseline to end of treatment, as measured by the BPI severity sub-scale. This threshold was chosen based on previous randomised controlled trials of duloxetine in osteoarthritis.

For peer review only

Primary outcomes

Treatment mechanism

(1) Change in the regional response of blood oxygenation level dependent (BOLD) contrast to pinprick nociceptive stimulation after duloxetine treatment (MRI2) vs. baseline (MRI1) in treatment responder group;

(2) Functional connectivity change from baseline (MRI1) to post duloxetine treatment (MRI2) using network metrics of BOLD fMRI under resting condition in treatment responder group;

Response prediction

(3) Differences in nociceptive BOLD response and network function at baseline between responders and non-responders;

Secondary outcomes

1. Difference in the regional response of blood oxygenation level dependent (BOLD) contrast to pinprick nociceptive stimulation after duloxetine treatment (MRI2) between responder and non-responder groups;
2. Functional connectivity differences post duloxetine treatment (MRI2) using network metrics of BOLD fMRI under resting condition between treatment responder and non-responder groups;
3. Correlation between baseline CPM and TS with brain activity and connectivity changes from baseline to post 6 weeks of duloxetine treatment (rsfMRI and pinprick fMRI).
4. Group differences in brain activity (pinprick fMRI and rsfMRI) and structure (T1-weighted brain MRI) in pain processing, limbic and modulatory pathways changes

For peer review only

from baseline to following 6 weeks of duloxetine treatment, in comparison to placebo.

5. Identification of baseline QST (CPM, TS, PPT) and questionnaire parameters (BDI, STAI, PCS, ICOAP) that predict response to duloxetine.
6. Multivariate treatment response prediction model.

Other outcomes

Exploratory outcomes include, but are not limited to, determination of gene variations that can be linked with duloxetine treatment response and GABA modulation according to changes in pain severity. Imaging data from the baseline study may be pooled with other studies undertaken in the Nottingham ARUK Pain Centre if the participants consent to this.

Data analysis

Clinical, QST and psychometric data will be compared between groups and conditions using the latest versions of statistical and image analysis software including SPSS (IBM).

Brain imaging data will be analysed using the latest versions of several established toolboxes: FSL (FMRIB),[27] PyMVPA,[28] SPM (Statistical Parametric Mapping version 8, London, UK), REST, Brain Connectivity Toolbox and GRETNA,[29-31] and LCmodel for MRS data.[32]. All data will undergo established quality control and standard recommended preprocessing. Preprocessing steps for functional data include motion correction, slice-timing correction using Fourier-space time-series phase-shifting, non-brain tissue removal, spatial co-registration to T1-weighted image and normalisation to the MNI anatomical standard space. Spatial smoothing will be carried out using an isotropic Gaussian kernel and a highpass temporal filtering will be applied. Next, for

For peer review only

task-related fMRI, statistical maps will be generated for contrasts of interest, such as to identify brain activity evoked by pinprick in each individual scan using first-level general linear model.

Z-scores will be extracted from regions of interest of pain processing nodes defined from meta-analysis,[6] emotion regulatory, default mode and salience network including anterior cingulate cortex, anterior insula, amygdala, brainstem and thalamus for both task-related and resting functional maps. Individual functional connectivity maps will be also generated. These will be used on higher level analysis which will include, among others: 1) Paired t-test to evaluate treatment effects; 2) two-sample t-test to compare groups (responders and non-responders) at baseline; 3) correlation analysis to determine associations with psychometric and QST measurements.

Sample size estimation

The sample size calculation was performed according to the guidelines stated by Lenth and colleagues.[33] Estimates of change in functional activation were deduced from a duloxetine intervention study (effect size was calculated based on p-values provided for the ACC).[16] Using the Java applets [34] it was estimated that a one-sample, one-tailed, t-test would have 80% power at alpha of <0.005 (to allow for ten multiple independent tests) with approximately 19 participants per group: responders to duloxetine treatment, non-responders, and placebo groups.

Based on previous experience of non-usable imaging data due to patient and / or technical factors in 10% of cases, a sample size of >22 participants per group was necessary. A further allowance for 20% dropout gives 27 participants in each group, resulting in a recruitment target of 81 participants.

For peer review only

Recruitment

Identification of potential participants is by contacting people who have taken part in previous studies within the Arthritis Research UK Pain Centre (ARUKPC). Recruitment through primary care and posters is also being used. Candidates are pre-screened during phone call following expression of interest. Medical notes are verified for eligibility following oral consent. Suitable candidates are then booked in for the first visit when written informed consent is obtained from all individuals taking part in the study by one of the investigators, before any interventions related to the study will take place. The procedure consists of the following: the investigator explains the details of the study and provides participant information sheet and drug information leaflet, ensuring that the participant has sufficient time to consider participating or not. The investigator then answers any questions that the participant may have concerning study participation, before signing the consent form.

Concomitant medication

Participants are instructed not to change their regular pain treatment while taking part in the study. Rescue pain medication, taken on an 'as needed basis', is accepted during the trial period and its use is documented in a daily pain diary.

Compliance

Compliance will be assessed in the first instance by their attendance at all scheduled research visits. Participants who choose to discontinue are asked to inform the investigators on their decision. Participants are contacted via telephone while undertaking the study medication in order to promote participant retention and completion of follow-up. During the second visit, participants are asked whether they took the drug according to the protocol.

For peer review only

Should the participant be unwilling to reschedule their appointment, data collected up until that point will be carried forward to the analysis stages and they will be withdrawn from the investigation. If a participant is unable to return for their second visit, follow-up questionnaires will obtained by post.

Criteria for withdrawal

Participation in this study is voluntary and participants are free to withdraw from the trial at any time, without giving any reason. However they must seek advice from the researchers before doing so as discontinuing duloxetine treatment suddenly may cause drug withdrawal effects. Involvement may be also discontinued by the investigators in case of any significant adverse effect of duloxetine/placebo treatment, or when subjects require major changes to their ongoing pain management, during the course of the study.

Adverse events and data safety monitoring

All observed or reported adverse events (AEs) are recorded by the investigators. No AEs are expected in relation to the MRI scanning. Potential AEs are anticipated to be only related to drug intake. All adverse events are recorded and monitored until resolution, stabilisation, or until it has been shown that the study intervention is not the cause. Any serious adverse event (SAE) is recorded and reported to the research ethics committee (REC) as part of the annual reports.

Handling and storage of data and documents

All study staff and investigators endeavour to protect the rights of the trial participants to privacy and informed consent, and will adhere to the Data Protection Act, 1998. Personal data

For peer review only

is handled confidentially. Imaging, sample and questionnaire data are anonymised by assigning a unique participant identification code, containing the abbreviation of the study's name followed by a sequence number. The link between study identification code and individual's personal details is kept on the consent form and MR screening form.

Computer held data is kept securely and password protected. All data is stored on a secure dedicated server. Access to data is given to the investigators and is restricted by user identifiers and passwords. Copies of the raw imaging data is also stored on CD/DVD in a locked room in the Radiological Sciences premises as back-up data. Either double data entry or checks of 40% of data entry are to be performed in order to promote data quality.

Direct access is permitted when required to all source documents and other study documentation e.g. signed consent forms, for the purpose of study monitoring and audit and other lawful regulatory inspection by the chief investigator, sponsor's designee and inspection by relevant regulatory authorities.

The investigators have full access to the dataset without limiting contractual obligations.

Monitoring trial conduct

Trial conduct is subject to systems audit of the Trial Master File for inclusion of essential documents, permissions to conduct the trial, trial delegation log, curriculum vitae of trial staff and training received, local document control procedures, consent procedures and recruitment logs, adherence to procedures defined in the protocol (e.g. inclusion / exclusion criteria, correct randomisation, timeliness of visits), adverse event recording and reporting, drug accountability, pharmacy records and equipment calibration logs.

The trial coordinator or a nominated designee of the sponsor shall carry out a site systems audit according to local requirements.

For peer review only

Ethics and dissemination

The study was approved by the East Midlands – Nottingham 2 research ethics committee (18/EM/0189) in August 2014 and is being carried out under the principles of the Declaration of Helsinki (64th, 2013) and Good Clinical Practice standards. Any modifications to the original protocol must undergo review by the research ethics committee involved. The design, documentation and data collection have been additionally discussed and shaped according to opinion of a focus group formed by representatives of patients and the public. The first participant was recruited in December 2014 and the last follow-up visit is expected in September 2016. Data analysis will start in October 2016. Results will be disseminated in peer-reviewed journals and at scientific meetings and conferences, where patient data will be presented anonymously.

Funding

This work is supported by Arthritis Research UK (Grant 18769) and the University of Nottingham. The investigators have full access to the final dataset without limiting contractual agreements.

Study sponsor: University of Nottingham

Sponsor's reference: 13124

Contact name: Angela Shone

Address: Room B50, King's Meadow Campus, University of Nottingham, Lenton Lane, Nottingham, NG7 2NR, UK

Telephone: 0115 8467906

Email: sponsor@nottingham.ac.uk

For peer review only

Contributors

DPA and DAW designed the study and DR, DPA, RDK, CRT and RPM developed the research protocol. DR leads the study, is responsible for data acquisition and will conduct image and statistical analyses. WC will undertake image and statistical analysis. CRT provides statistical consultation. DR, HB and WC undertake recruitment and data collection. DR and DPA wrote the manuscript, which was edited by CRT and RDK. All authors approved the final manuscript and authorship eligibility will follow guidelines of biomedical journals.

Competing interests

The authors have no potential conflicts of interest with respect to the research, authorship and publication of this article.

References

1. McAlindon TE, Bannuru RR, Sullivan M, et al. OARSI guidelines for the non-surgical management of knee osteoarthritis. *Osteoarthritis and cartilage* 2014;22(3):363-88.
2. Beswick AD, Wylde V, Gooberman-Hill R, et al. What proportion of patients report long-term pain after total hip or knee replacement for osteoarthritis? A systematic review of prospective studies in unselected patients. *BMJ open* 2012;2(1):e000435.
3. Ohtori S, Orita S, Yamashita M, et al. Existence of a neuropathic pain component in patients with osteoarthritis of the knee. *Yonsei medical journal* 2012;53(4):801-05.
4. Sanders D, Krause K, O'Muircheartaigh J, et al. Pharmacologic Modulation of Hand Pain in Osteoarthritis: A Double-Blind Placebo-Controlled Functional Magnetic Resonance Imaging Study Using Naproxen. *Arthritis & Rheumatology* 2015;67(3):741-51.
5. Vachon-Presseau E, Tétreault P, Petre B, et al. Corticolimbic anatomical characteristics predetermine risk for chronic pain. *Brain* 2016:aww100.
6. Tanasescu R, Cottam WJ, Condon L, et al. Functional reorganisation in chronic pain and neural correlates of pain sensitisation: A coordinate based meta-analysis of 266 cutaneous pain fMRI studies. *Neuroscience & Biobehavioral Reviews* 2016;68:120-33.

7. Petzke F, Jensen K, Kosek E, et al. Using fMRI to evaluate the effects of milnacipran on central pain processing in patients with fibromyalgia. *Scandinavian Journal of Pain* 2013;4(2):65-74.
8. Giménez M, Pujol J, Ali Z, et al. Naproxen effects on brain response to painful pressure stimulation in patients with knee osteoarthritis: a double-blind, randomized, placebo-controlled, single-dose study. *The Journal of rheumatology* 2014;41(11):2240-48.
9. Duff EP, Vennart W, Wise RG, et al. Learning to identify CNS drug action and efficacy using multistudy fMRI data. *Science translational medicine* 2015;7(274):274ra16-74ra16.
10. Tracey I. Can neuroimaging studies identify pain endophenotypes in humans? *Nature Reviews Neurology* 2011;7(3):173-81.
11. Wager TD, Atlas LY, Lindquist MA, et al. An fMRI-based neurologic signature of physical pain. *New England Journal of Medicine* 2013;368(15):1388-97.
12. Brown GG, Perthen JE, Liu TT, et al. A primer on functional magnetic resonance imaging. *Neuropsychology review* 2007;17(2):107-25.
13. Petersen E, Zimine I, Ho YL, et al. Non-invasive measurement of perfusion: a critical review of arterial spin labelling techniques. *The British journal of radiology* 2014
14. Chappell AS, Ossanna MJ, Liu-Seifert H, et al. Duloxetine, a centrally acting analgesic, in the treatment of patients with osteoarthritis knee pain: a 13-week, randomized, placebo-controlled trial. *Pain* 2009;146(3):253-60.
15. Chappell AS, Desaiah D, Liu-Seifert H, et al. A double-blind, randomized, placebo-controlled study of the efficacy and safety of duloxetine for the treatment of chronic pain due to osteoarthritis of the knee. *Pain Practice* 2011;11(1):33-41.
16. Van Marle HJ, Tendolkar I, Urner M, et al. Subchronic duloxetine administration alters the extended amygdala circuitry in healthy individuals. *Neuroimage* 2011;55(2):825-31.
17. Fu CH, Costafreda SG, Sankar A, et al. Multimodal functional and structural neuroimaging investigation of major depressive disorder following treatment with duloxetine. *BMC psychiatry* 2015;15(1):1.
18. Freynhagen R, Baron R, Gockel U, et al. Pain DETECT: a new screening questionnaire to identify neuropathic components in patients with back pain. *Current medical research and opinion* 2006;22(10):1911-20.
19. Sullivan MJ, Bishop SR, Pivik J. The pain catastrophizing scale: development and validation. *Psychological assessment* 1995;7(4):524.
20. Spielberger CD. State-Trait anxiety inventory: Wiley Online Library 2010.
21. Beck AT, Steer RA, Brown GK. Beck depression inventory-II. *San Antonio, TX* 1996:78204-2498.
22. Hawker G, Davis A, French M, et al. Development and preliminary psychometric testing of a new OA pain measure–an OARSI/OMERACT initiative. *Osteoarthritis and Cartilage* 2008;16(4):409-14.
23. Ayearst LE, Harsanyi Z, Michalko KJ. The Pain and Sleep Questionnaire three-item index (PSQ-3): A reliable and valid measure of the impact of pain on sleep in chronic

For peer review only

nonmalignant pain of various etiologies. *Pain Research and Management* 2012;17(4):281-90.

24. Cleeland C, Ryan K. Pain assessment: global use of the Brief Pain Inventory. *Annals of the Academy of Medicine, Singapore* 1994;23(2):129-38.
25. Borson S, Scanlan J, Brush M, et al. The Mini-Cog: a cognitive'vital signs' measure for dementia screening in multi-lingual elderly. *International journal of geriatric psychiatry* 2000;15(11):1021-27.
26. Pouli N, Nair RD, Lincoln NB, et al. The experience of living with knee osteoarthritis: exploring illness and treatment beliefs through thematic analysis. *Disability and rehabilitation* 2014
27. Jenkinson M, Beckmann CF, Behrens TE, et al. Fsl. *Neuroimage* 2012;62(2):782-90.
28. Hanke M, Halchenko YO, Sederberg PB, et al. PyMVPA: a unifying approach to the analysis of neuroscientific data. *Python in Neuroscience* 2015:157.
29. Song X-W, Dong Z-Y, Long X-Y, et al. REST: a toolkit for resting-state functional magnetic resonance imaging data processing. *PloS one* 2011;6(9):e25031.
30. Wang J, Wang X, Xia M, et al. GRETNA: a graph theoretical network analysis toolbox for imaging connectomics. *Frontiers in human neuroscience* 2015;9
31. Rubinov M, Sporns O. Complex network measures of brain connectivity: uses and interpretations. *Neuroimage* 2010;52(3):1059-69.
32. Provencher SW. Estimation of metabolite concentrations from localized in vivo proton NMR spectra. *Magnetic resonance in medicine* 1993;30(6):672-79.
33. Lenth RV. Some practical guidelines for effective sample size determination. *The American Statistician* 2001;55(3):187-93.
34. Lenth RV. Java applets for power and sample size [computer software]. *Retrieved October* 2006;31:2013.

For peer review only

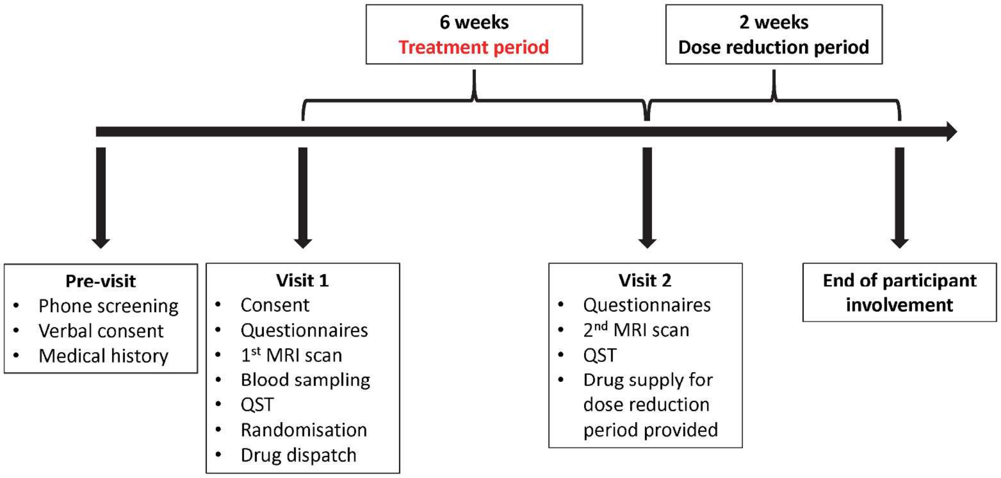

Participation timeline and general characteristics of each study visit. QST, quantitative sensory testing; MRI, magnetic resonance imaging
(Insert Figure 1)
251x120mm (300 x 300 DPI)

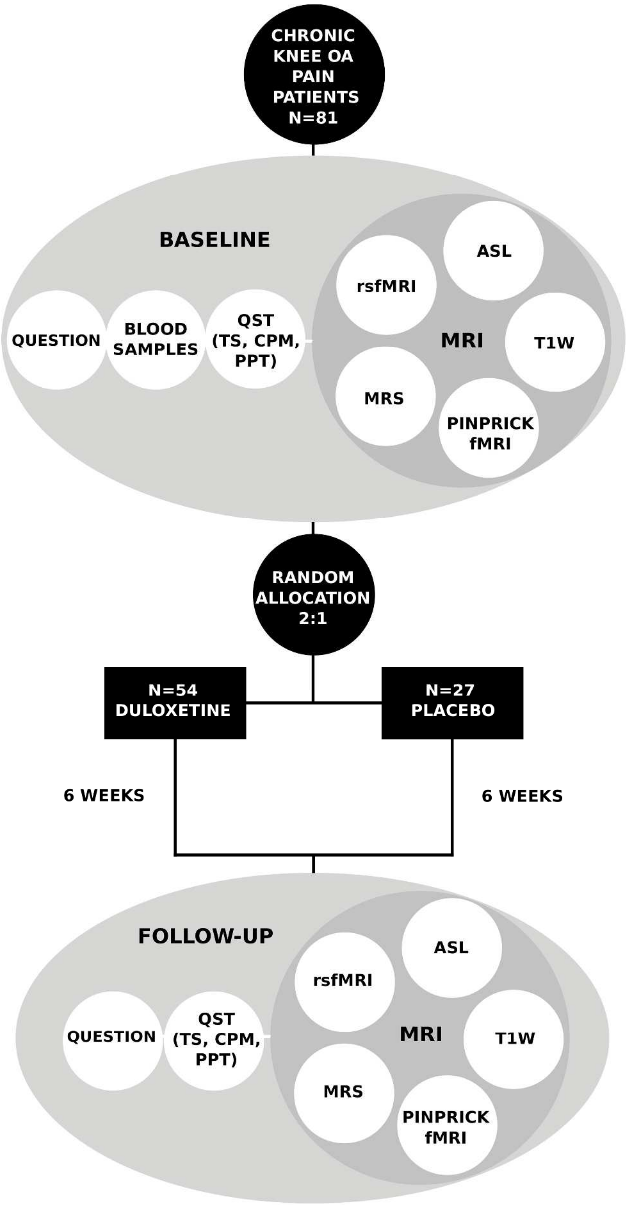

Details of experimental testing and randomisation of participants. Volume of interest for MRS is located in the mid anterior cingulate cortex. OA, osteoarthritis; QST, quantitative sensory testing; TS, temporal summation; PPT, pressure pain thresholds; CPM, conditioned pain modulation; Question, questionnaires; MRI, magnetic resonance imaging; fMRI, functional magnetic resonance imaging; rsfMRI, resting state fMRI; ASL, arterial spin labelling; T1w, T1-weighted MRI.
(Insert Figure 2)
330x634mm (300 x 300 DPI)

SPIRIT

STANDARD PROTOCOL ITEMS: RECOMMENDATIONS FOR INTERVENTIONAL TRIALS

SPIRIT 2013 Checklist: Recommended items to address in a clinical trial protocol and related documents*

Section/item	Item No	Description	Addressed on page number
**Administrative information**			
Title	1	Descriptive title identifying the study design, population, interventions, and, if applicable, trial acronym	______1______
Trial registration	2a	Trial identifier and registry name. If not yet registered, name of intended registry	______2______
	2b	All items from the World Health Organization Trial Registration Data Set	______ok_____
Protocol version	3	Date and version identifier	______NA______
Funding	4	Sources and types of financial, material, and other support	______2______
Roles and responsibilities	5a	Names, affiliations, and roles of protocol contributors	______1,16___
	5b	Name and contact information for the trial sponsor	__18, 19______
	5c	Role of study sponsor and funders, if any, in study design; collection, management, analysis, and interpretation of data; writing of the report; and the decision to submit the report for publication, including whether they will have ultimate authority over any of these activities	_____10_______
	5d	Composition, roles, and responsibilities of the coordinating centre, steering committee, endpoint adjudication committee, data management team, and other individuals or groups overseeing the trial, if applicable (see Item 21a for data monitoring committee)	_____NA_____

For peer review only - http://bmjopen.bmj.com/site/about/guidelines.xhtml

Introduction

Background and rationale	6a	Description of research question and justification for undertaking the trial, including summary of relevant studies (published and unpublished) examining benefits and harms for each intervention	3,4,5,6
	6b	Explanation for choice of comparators	
Objectives	7	Specific objectives or hypotheses	5,6,7
Trial design	8	Description of trial design including type of trial (eg, parallel group, crossover, factorial, single group), allocation ratio, and framework (eg, superiority, equivalence, noninferiority, exploratory)	7,10

Methods: Participants, interventions, and outcomes

Study setting	9	Description of study settings (eg, community clinic, academic hospital) and list of countries where data will be collected. Reference to where list of study sites can be obtained	7
Eligibility criteria	10	Inclusion and exclusion criteria for participants. If applicable, eligibility criteria for study centres and individuals who will perform the interventions (eg, surgeons, psychotherapists)	7,8,9
Interventions	11a	Interventions for each group with sufficient detail to allow replication, including how and when they will be administered	10
	11b	Criteria for discontinuing or modifying allocated interventions for a given trial participant (eg, drug dose change in response to harms, participant request, or improving/worsening disease)	15
	11c	Strategies to improve adherence to intervention protocols, and any procedures for monitoring adherence (eg, drug tablet return, laboratory tests)	
	11d	Relevant concomitant care and interventions that are permitted or prohibited during the trial	14
Outcomes	12	Primary, secondary, and other outcomes, including the specific measurement variable (eg, systolic blood pressure), analysis metric (eg, change from baseline, final value, time to event), method of aggregation (eg, median, proportion), and time point for each outcome. Explanation of the clinical relevance of chosen efficacy and harm outcomes is strongly recommended	12,13
Participant timeline	13	Time schedule of enrolment, interventions (including any run-ins and washouts), assessments, and visits for participants. A schematic diagram is highly recommended (see Figure)	9,10,11+Fig1

For peer review only - http://bmjopen.bmj.com/site/about/guidelines.xhtml

Sample size	14	Estimated number of participants needed to achieve study objectives and how it was determined, including clinical and statistical assumptions supporting any sample size calculations	______14______
Recruitment	15	Strategies for achieving adequate participant enrolment to reach target sample size	______14_____

Methods: Assignment of interventions (for controlled trials)

Allocation:			
Sequence generation	16a	Method of generating the allocation sequence (eg, computer-generated random numbers), and list of any factors for stratification. To reduce predictability of a random sequence, details of any planned restriction (eg, blocking) should be provided in a separate document that is unavailable to those who enrol participants or assign interventions	______10_____
Allocation concealment mechanism	16b	Mechanism of implementing the allocation sequence (eg, central telephone; sequentially numbered, opaque, sealed envelopes), describing any steps to conceal the sequence until interventions are assigned	_____10______
Implementation	16c	Who will generate the allocation sequence, who will enrol participants, and who will assign participants to interventions	_____10______
Blinding (masking)	17a	Who will be blinded after assignment to interventions (eg, trial participants, care providers, outcome assessors, data analysts), and how	_____10______
	17b	If blinded, circumstances under which unblinding is permissible, and procedure for revealing a participant's allocated intervention during the trial	_____10______

Methods: Data collection, management, and analysis

Data collection methods	18a	Plans for assessment and collection of outcome, baseline, and other trial data, including any related processes to promote data quality (eg, duplicate measurements, training of assessors) and a description of study instruments (eg, questionnaires, laboratory tests) along with their reliability and validity, if known. Reference to where data collection forms can be found, if not in the protocol	____10,11,12___
	18b	Plans to promote participant retention and complete follow-up, including list of any outcome data to be collected for participants who discontinue or deviate from intervention protocols	______15,16____

Data management	19	Plans for data entry, coding, security, and storage, including any related processes to promote data quality (eg, double data entry; range checks for data values). Reference to where details of data management procedures can be found, if not in the protocol	______17_______
Statistical methods	20a	Statistical methods for analysing primary and secondary outcomes. Reference to where other details of the statistical analysis plan can be found, if not in the protocol	_____13,14____
	20b	Methods for any additional analyses (eg, subgroup and adjusted analyses)	____13,14______
	20c	Definition of analysis population relating to protocol non-adherence (eg, as randomised analysis), and any statistical methods to handle missing data (eg, multiple imputation)	______13______
**Methods: Monitoring**			
Data monitoring	21a	Composition of data monitoring committee (DMC); summary of its role and reporting structure; statement of whether it is independent from the sponsor and competing interests; and reference to where further details about its charter can be found, if not in the protocol. Alternatively, an explanation of why a DMC is not needed	_____NA_______
	21b	Description of any interim analyses and stopping guidelines, including who will have access to these interim results and make the final decision to terminate the trial	_____NA______
Harms	22	Plans for collecting, assessing, reporting, and managing solicited and spontaneously reported adverse events and other unintended effects of trial interventions or trial conduct	_____15,16_____
Auditing	23	Frequency and procedures for auditing trial conduct, if any, and whether the process will be independent from investigators and the sponsor	______17_______
**Ethics and dissemination**			
Research ethics approval	24	Plans for seeking research ethics committee/institutional review board (REC/IRB) approval	_____2, 16____
Protocol amendments	25	Plans for communicating important protocol modifications (eg, changes to eligibility criteria, outcomes, analyses) to relevant parties (eg, investigators, REC/IRBs, trial participants, trial registries, journals, regulators)	_____16______

For peer review only - http://bmjopen.bmj.com/site/about/guidelines.xhtml

Consent or assent	26a	Who will obtain informed consent or assent from potential trial participants or authorised surrogates, and how (see Item 32)	____15_______
	26b	Additional consent provisions for collection and use of participant data and biological specimens in ancillary studies, if applicable	____14________
Confidentiality	27	How personal information about potential and enrolled participants will be collected, shared, and maintained in order to protect confidentiality before, during, and after the trial	____16_______
Declaration of interests	28	Financial and other competing interests for principal investigators for the overall trial and each study site	____16,17_____
Access to data	29	Statement of who will have access to the final trial dataset, and disclosure of contractual agreements that limit such access for investigators	____17_______
Ancillary and post-trial care	30	Provisions, if any, for ancillary and post-trial care, and for compensation to those who suffer harm from trial participation	____ok________
Dissemination policy	31a	Plans for investigators and sponsor to communicate trial results to participants, healthcare professionals, the public, and other relevant groups (eg, via publication, reporting in results databases, or other data sharing arrangements), including any publication restrictions	____18_________
	31b	Authorship eligibility guidelines and any intended use of professional writers	____16_______
	31c	Plans, if any, for granting public access to the full protocol, participant-level dataset, and statistical code	____NA_______
**Appendices**			
Informed consent materials	32	Model consent form and other related documentation given to participants and authorised surrogates	_____ok_______
Biological specimens	33	Plans for collection, laboratory evaluation, and storage of biological specimens for genetic or molecular analysis in the current trial and for future use in ancillary studies, if applicable	____ok_______

*It is strongly recommended that this checklist be read in conjunction with the SPIRIT 2013 Explanation & Elaboration for important clarification on the items. Amendments to the protocol should be tracked and dated. The SPIRIT checklist is copyrighted by the SPIRIT Group under the Creative Commons "Attribution-NonCommercial-NoDerivs 3.0 Unported" license.

For peer review only - http://bmjopen.bmj.com/site/about/guidelines.xhtml

BMJ Open

Imaging Pain Relief in Osteoarthritis (IPRO): protocol of a double blind randomised controlled mechanistic study assessing pain relief and prediction of duloxetine treatment outcome

For peer review only

Journal:	*BMJ Open*
Manuscript ID	bmjopen-2016-014013.R2
Article Type:	Protocol
Date Submitted by the Author:	02-Feb-2017
Complete List of Authors:	Reckziegel, Diane; University of Nottingham, Arthritis Research UK Pain Centre; University of Nottingham, Sir Peter Mansfield Imaging Centre<br>Bailey, Helen; University of Nottingham, Arthritis Research UK Pain Centre; University of Nottingham, Sir Peter Mansfield Imaging Centre<br>Cottam, William; University of Nottingham, Arthritis Research UK Pain Centre; University of Nottingham, Sir Peter Mansfield Imaging Centre<br>Tench, Christopher; University of Nottingham, Division of Clinical Neuroscience<br>Mahajan, Ravi; University of Nottingham, Anaesthesia and Intensive Care<br>Walsh, David; University of Nottingham, Academic Rheumatology<br>Knaggs, Roger; University of Nottingham, School of Pharmacy<br>Auer, Dorothee; University of Nottingham, Arthritis Research UK Pain Centre; University of Nottingham, Sir Peter Mansfield Imaging Centre
**Primary Subject Heading**:	Radiology and imaging
Secondary Subject Heading:	Pharmacology and therapeutics
Keywords:	osteoarthritis pain, analgesia, duloxetine, antidepressant, functional magnetic resonance imaging, MRI

SCHOLARONE™ Manuscripts

Study protocol

Corresponding author:

Dorothee P Auer, Radiological Sciences, Room W/B 1441, Queen's Medical Centre, Nottingham, NG72UH, UK.

Email: Dorothee.Auer@nottingham.ac.uk

Imaging Pain Relief in Osteoarthritis (IPRO): protocol of a double blind randomised controlled mechanistic study assessing pain relief and prediction of duloxetine treatment outcome

Diane Reckziegel[a,b,c], Helen Bailey[a,b,c], William J Cottam[a,b,c], Christopher R Tench[d], Ravi P Mahajan[a,e], David A Walsh[a,f], Roger D Knaggs[a,g], Dorothee P Auer[a,b,c]

[a]*Arthritis Research UK Pain Centre, University of Nottingham, UK.* [b]*Sir Peter Mansfield Imaging Centre, University of Nottingham, UK.* [c]*Division of Clinical Neuroscience, Radiological Sciences, University of Nottingham, UK.* [d]*Division of Clinical Neuroscience, Clinical Neurology, University of Nottingham, UK.* [e]*Division of Clinical Neuroscience, Anaesthesia and Critical Care, University of Nottingham, UK .*[f]*Division of Rheumatology, Orthopaedics and Dermatology, University of Nottingham, UK.* [g]*School of Pharmacy, University of Nottingham, UK*

We acknowledge other researchers, who significantly contribute to recruitment (Nadia Frowd), help with data collection (Marianne Drabek) and contributed to the initial local internal application (Naranjargal Dashdorj). This work was supported by Arthritis Research UK (Grant 18769) and the University of Nottingham.

For peer review only

Abstract

Introduction: Osteoarthritis (OA) pain is a major cause of long-term disability and chronic pain in the adult population. One in five patients does not receive satisfactory pain relief, which reflects the complexity of chronic pain and the current lack of understanding of mechanisms of chronic pain. Recently, duloxetine has demonstrated clinically relevant pain relief, but only in half of treated patients with OA. Here the aim is to investigate the neural mechanisms of pain relief and neural signatures that may predict treatment response to duloxetine in chronic knee OA pain.

Methods and analysis: This is an ongoing single-centre randomised placebo-controlled mechanistic study (2:1 [placebo] allocation), using a multi-modal neuroimaging approach, together with psychophysiological [quantitative sensory testing], genetics and questionnaire assessments. Eighty-one subjects with chronic knee OA pain are planned to power for between group comparisons (placebo, duloxetine-responder, and duloxetine-non-responder). Participants have a baseline assessment and, following six weeks of duloxetine (30mg for two weeks, then 60mg for four weeks), a follow-up evaluation. Brain imaging is performed at 3T with blood-oxygen-level dependent functional magnetic resonance imaging at rest and during pin-prick nociceptive stimulation for main outcome assessment; arterial spin labelling and structural imaging (T1-weighted) for secondary outcome assessment. Questionnaires evaluate pain, negative affect, quality of sleep and cognition.

Ethics and dissemination: The study has been approved by the East Midlands – Nottingham 2 research ethics committee (18/EM/0189) and is being carried out under the principles of the Declaration of Helsinki (64th, 2013) and Good Clinical Practice standards. Results will be disseminated in peer-reviewed journals and at scientific conferences.

Trial registration number: This trial is registered at ClinicalTrials.gov (NCT02208778).

This work was supported by Arthritis Research UK (Grant 18769).

Keywords: Osteoarthritis pain, magnetic resonance imaging, fMRI, analgesia, duloxetine, antidepressant

For peer review only

Strengths and limitations of the study

- This study attempts to shed light on the little understood mechanisms of pain relief in chronic pain.
- By undertaking an innovative and integrative approach, which combines multi-modal neuroimaging, quantitative sensory testing and psychometric questionnaire assessments, drug effects and predictors of treatment response to duloxetine are aimed to be identified.
- A cross-over design would have ideally been employed. However, a parallel design was chosen in order to minimise dropout rates.

Background and rationale

Osteoarthritis (OA) is a major cause of long-term disability and chronic pain in the adult population. Knee and hip OA combined ranked 11th in a recent survey of global burden of disease, and symptomatic knee OA is several fold more common than hip OA (.[1] Current treatment options consist of a combination of pharmacological and non-pharmacological alternatives.[2] Despite these options, around 20% of patients do not get satisfactory pain relief, even after undergoing joint replacement surgery.[3] This strongly supports the notion that chronic osteoarthritis pain has a strong central component[4-6]

Application of advanced neuroimaging tools led to novel understanding of central mechanisms underpinning acute and chronic pain states.[7] Functional imaging in particular lends itself to the mechanistic study of analgesic treatment.[5 8-10] Neuroimaging may also prove useful in guiding mechanism-based personalised therapy.[10-12]

To comprehensively describe the brain‘s activation state across regions and functional properties, the concept of so-called brain ‘signatures’ has emerged that can be derived from

For peer review only

regional and network analysis of blood oxygen level dependent (BOLD) functional MRI data at rest or during controlled stimulation, or from cerebral blood flow maps based on arterial spin labelling (ASL) techniques.[13 14]

Duloxetine, a serotonin and noradrenaline reuptake inhibitor, has antidepressant and anxiolytic properties and is currently also used to treat chronic pain conditions. However, the precise mechanisms by which this drug acts to relieve pain are unclear and only around 50% of OA patients have sufficient pain relief after duloxetine intake;[15] currently there is no way to predict responders.[15 16] In healthy participants, duloxetine was found to attenuate activity in affect processing areas (amygdala, thalamus, insula and ACC) while simultaneously increasing functional coupling between the amygdala and the anterior insula, during an emotional face matching task.[17] Duloxetine in major depression augmented connectivity in the anterior *default mode network (DMN)*,[18] while reduced connectivity within the subgenual cingulate was predictive of clinical antidepressant response.[18]

The aim of this study is to investigate the neural mechanisms of pain relief in chronic osteoarthritis pain following treatment with duloxetine, and to establish multimodal brain signatures to predict treatment response. We will use a unique multimodal MRI design to identify key mechanisms and predictors of duloxetine-induced pain relief in chronic OA pain. This will include brain resting state functional connectivity (rsfc), ASL, nociceptive fMRI, structural T1-weighted brain MRI, proton spectroscopy, quantitative sensory testing (QST) and questionnaire assessments.

We hypothesise that responding and non-responding OA patients have different limbic signatures, differences in negative affect and sensory pain phenotypes. Additionally, we hypothesise that duloxetine induces functional brain changes in emotional regulatory networks.

For peer review only

Primary objectives

To identify a functional change in brain signature indexing the analgesic mechanism of duloxetine.

To identify brain imaging and psychophysical markers that predict the response to duloxetine treatment.

Main hypotheses

- Analgesic response to duloxetine treatment can be predicted using a range of baseline brain imaging markers and QST.
- Analgesic response to duloxetine is mediated by modulation of neural networks underpinning emotional control.
- Duloxetine-induced changes in brain activation differ between responders and non-responders.

Design and Methods

This is a single centre, double-blind, placebo controlled mechanistic study being conducted at the University of Nottingham (United Kingdom).

Inclusion criteria

In order to participate in this study, individuals must have chronic knee pain with radiographically defined OA changes (Kellgren Lawrence [KL) grade 2 or higher), be able to

For peer review only

consent to participation and be aged 35 or older. They must also be free of major medical, neurological and psychiatric comorbidities known to affect neural processing of pain.

Exclusion criteria

Potential candidates meeting any of the criteria listed below are excluded from participation.

General:

- Significant medical conditions, including uncontrolled hypertension, insulin dependent diabetes, dementia, Parkinson's disease, epilepsy, multiple sclerosis, active cancer, major depressive disorder, mania, bipolar and schizophrenia. This is judged by a qualified physician based on medical records;
- Refusal by candidate to GP being informed;
- Planned total knee replacement;

MRI-related:

- Intraocular metallic foreign bodies;
- Intracranial aneurysm clips;
- Cardiac pacemakers and defibrillators;
- Cochlear implants;
- Significant head tremor;
- Potential metal foreign bodies due to previous accidents;
- Breastfeeding or pregnancy, confirmed by pregnancy test;

For peer review only

- Unfitness for the MRI scanner, according to the judgement of medically qualified personnel, either on the research team, or the patient's clinical team (*e.g.* due to back pain, claustrophobia, acute sickness etc). This includes patients with signs of impaired temperature regulation such as an extremely high fever;
- Patients with large tattoos, specifically in the head, neck or shoulder region;
- Metallic agents embedded within the body (*i.e.* Shrapnel, surgical pins);

Duloxetine-related:

- Uncontrolled narrow-angle glaucoma;
- Recent usage of monoamine oxidase inhibitor (MAOI) or Mellaril® (thioridazine);
- Taking St. John's Wort (Hypericum perforatum);
- On fluvoxamine, ciprofloxacin or enoxacin;
- Taking other medicines containing duloxetine;
- Liver disease;
- Renal impairment;
- Currently on antidepressant treatment, including treatment for pain with tricyclic agents such as amitriptyline;
- Taking tramadol;
- Known hypersensitivity, allergy or intolerance to one of duloxetine's components;
- Unwillingness to take caution in relation to use of other centrally active substances such as alcohol and sedative drugs;
- Current treatment with potent inhibitors of CYP1A2 like fluvoxamine;

For peer review only

- History of seizures or any drug lowering the seizure threshold

Randomisation and blinding

Participants are randomised between duloxetine (n=54) and placebo (n=27). The allocation ratio is 2:1; given that around half of the population undergoing active treatment is expected to have satisfactory pain relief, this should result in approximately equal numbers between groups of placebo intakes, responders and non-responders. A block randomisation, with block sizes of 9 and 2, is utilised, and was generated by the Clinical Trials Pharmacy (CTP) at Nottingham University Hospitals (NHS Trust) using a pseudo-random algorithm at http://www.randomization.com. The allocation list is kept by the CTP in a password-protected file. Investigators may identify the treatment for each participant via request to the CTP, however, during treatment, this breaking the code procedure may only be done in the event of a medical emergency, when treatment is dependent on knowledge of the actual drug received. In case the treatment code for a participant is broken or other actions, such as recording date or reasons for breaking the blind, discontinuation of trial treatment must be reported to the sponsor (University of Nottingham).

(Insert Figure 1)

Participants are assigned, by the investigators, a unique identification number (sequentially numbered) and attend the university twice, for both a baseline and follow-up visits. Treatment consists of six weeks of taking either duloxetine capsules or placebo equivalent, followed by two weeks of dose reduction for those taking duloxetine, in order to minimise drug withdrawal effects (Figure 1). In order to minimise potential side effects, participants take one tablet (duloxetine 30mg) daily during two weeks prior to switching to the target dose of 60mg/day.

Both researchers and participants are blinded to the treatment allocation until data acquisition is complete. At the end of the follow-up visit, individual allocation is disclosed to the participant only, by one of the CTP staff members, who are not involved in the any data analysis or research procedures. Data analysts will be blinded to treatment allocation.

Individual participation in the study is for around eight weeks, starting with signing informed consent and ending with the conclusion of the drug reduction phase.

(Insert Figure 2)

Experimental testing

The experimental testing is primarily based on multi-modal neuroimaging and QST, in conjunction with questionnaire assessment and genetics. This should provide a broad evaluation of the multi-faceted nature of pain, assessing both central and peripheral mechanisms, as well as psychological and cognitive traits, among others, which should allow pinpointing precise phenotyping of patients into distinct groups. Time points and types of data being collected are summarised in Figure 2 and details of questionnaires are given below.

Questionnaires:

- PainDetect (baseline) – a screening questionnaire to identify neuropathic components of pain;[19]
- Pain Catastrophizing Scale (PCS) (baseline) – a 13-item scale to measure pain catastrophising, which can be split into three sub-scales named rumination, magnification and helplessness;[20]

For peer review only

- State-Trait Anxiety Inventory (STAI) (baseline) – a psychological inventory assessing anxiety. It contains 40 items and can be split into two sub-scales being state anxiety and trait anxiety;[21]
- Beck Depression Inventory II (BDI) (baseline) – a 21-item multiple-choice self-report scale for measuring the severity of depression;[22]
- The intermittent and constant pain score (ICOAP) (baseline and follow-up) – an 11-question scale to characterise pain in individuals with osteoarthritis pain. It gives two sub-scales relating to intermittent and constant experience of pain;[23]
- The pain sleep questionnaire (baseline) – a 5-item tool to assess the impact of pain on sleep;[24]
- Brief Pain Inventory (BPI) (daily) – a 9-item questionnaire for measuring the severity of pain and the impact of this pain on daily functioning. The severity sub-scale is used to define the response definition (below);[25]
- Mini-Cog (baseline) – contains a 3-item recall test and a clock drawing test to screen cognitive impairment;[26]
- Educational Level (baseline);
- Questions on belief (baseline) – consist of four questions to assess an individual's expectations on treatment and medical care;[27]

Participant general details:

- Age;
- Sex;

For peer review only

- Pain intensity (measured on a numeric rating scale ranging from 0-100, where 0 represents no pain at all and 100 stands for excruciating pain);
- Pain duration;
- Analgesia intake (last 24h);
- Caffeine intake (last 24h);
- Average daily caffeine intake;
- Average weekly alcohol intake;
- Medication;

Other:

- 10ml blood samples;
- Analgesia diary (daily).

Response definition

Response to duloxetine is defined as a 50% reduction in the severity of ongoing pain from baseline to end of treatment, as measured by the BPI severity sub-scale. This threshold was chosen based on previous randomised controlled trials of duloxetine in osteoarthritis.

For peer review only

Primary outcomes

Treatment mechanism

(1) Change in the regional response of blood oxygenation level dependent (BOLD) contrast to pinprick nociceptive stimulation after duloxetine treatment (MRI2) vs. baseline (MRI1) in treatment responder group;

(2) Functional connectivity change from baseline (MRI1) to post duloxetine treatment (MRI2) using network metrics of BOLD fMRI under resting condition in treatment responder group;

Response prediction

(3) Differences in nociceptive BOLD response and network function at baseline between responders and non-responders;

Secondary outcomes

1. Difference in the regional response of blood oxygenation level dependent (BOLD) contrast to pinprick nociceptive stimulation after duloxetine treatment (MRI2) between responder and non-responder groups;
2. Functional connectivity differences post duloxetine treatment (MRI2) using network metrics of BOLD fMRI under resting condition between treatment responder and non-responder groups;
3. Correlation between baseline CPM and TS with brain activity and connectivity changes from baseline to post 6 weeks of duloxetine treatment (rsfMRI and pinprick fMRI).
4. Group differences in brain activity (pinprick fMRI and rsfMRI) and structure (T1-weighted brain MRI) in pain processing, limbic and modulatory pathways changes

For peer review only

from baseline to following 6 weeks of duloxetine treatment, in comparison to placebo.

5. Identification of baseline QST (CPM, TS, PPT) and questionnaire parameters (BDI, STAI, PCS, ICOAP) that predict response to duloxetine.
6. Multivariate treatment response prediction model.

Other outcomes

Exploratory outcomes include, but are not limited to, determination of gene variations that can be linked with duloxetine treatment response and GABA modulation according to changes in pain severity. Imaging data from the baseline study may be pooled with other studies undertaken in the Nottingham ARUK Pain Centre if the participants consent to this.

Data analysis

Clinical, QST and psychometric data will be compared between groups and conditions using the latest versions of statistical and image analysis software including SPSS (IBM).

Brain imaging data will be analysed using the latest versions of several established toolboxes: FSL (FMRIB),[28] PyMVPA,[29] SPM (Statistical Parametric Mapping version 8, London, UK), REST, Brain Connectivity Toolbox and GRETNA,[30-32] and LCModel for MRS data.[33]. All data will undergo established quality control and standard recommended preprocessing. Preprocessing steps for functional data include motion correction, slice-timing correction using Fourier-space time-series phase-shifting, non-brain tissue removal, spatial co-registration to T1-weighted image and normalisation to the MNI anatomical standard space. Spatial smoothing will be carried out using an isotropic Gaussian kernel and a high-pass temporal filtering will be applied. Next, for

For peer review only

task-related fMRI, statistical maps will be generated for contrasts of interest, such as to identify brain activity evoked by pinprick in each individual scan using first-level general linear model.

Z-scores will be extracted from regions of interest of pain processing nodes defined from meta-analysis,[7] emotion regulatory, default mode and salience network including anterior cingulate cortex, anterior insula, amygdala, brainstem and thalamus for both task-related and resting functional maps. Individual functional connectivity maps will be also generated. These will be used on higher level analysis which will include, among others: 1) Paired t-test to evaluate treatment effects; 2) two-sample t-test to compare groups (responders and non-responders) at baseline; 3) correlation analysis to determine associations with psychometric and QST measurements.

Sample size estimation

The sample size calculation was performed according to the guidelines stated by Lenth and colleagues.[34] Estimates of change in functional activation were deduced from a duloxetine intervention study (effect size was calculated based on p-values provided for the ACC).[17] Using the Java applets [35] it was estimated that a one-sample, one-tailed, t-test would have 80% power at alpha of <0.005 (to allow for ten multiple independent tests) with approximately 19 participants per group: responders to duloxetine treatment, non-responders, and placebo groups.

Based on previous experience of non-usable imaging data due to patient and / or technical factors in 10% of cases, a sample size of >22 participants per group was necessary. A further allowance for 20% dropout gives 27 participants in each group, resulting in a recruitment target of 81 participants. The study is not be powered to assess clinical efficacy.

For peer review only

Recruitment

Identification of potential participants is done by contacting people who have taken part in previous studies within the Arthritis Research UK Pain Centre (ARUKPC). Recruitment through primary care and posters is also being used. Candidates are pre-screened during phone call following expression of interest. Medical notes are verified for eligibility following oral consent. Suitable candidates are then booked in for the first visit when written informed consent is obtained from all individuals taking part in the study by one of the investigators, before any interventions related to the study will take place. The procedure consists of the following: the investigator explains the details of the study and provides participant information sheet and drug information leaflet, ensuring that the participant has sufficient time to consider participating or not. The investigator then answers any questions that the participant may have concerning study participation, before signing the consent form.

Concomitant medication

Participants are instructed not to change their regular pain treatment while taking part in the study. Rescue pain medication, taken on an 'as needed basis', is accepted during the trial period and its use is documented in a daily pain diary.

Compliance

Compliance will be assessed in the first instance by their attendance at all scheduled research visits. Participants who choose to discontinue are asked to inform the investigators on their decision. Participants are contacted via telephone while undertaking the study medication in order to promote participant retention and completion of follow-up. During the second visit, participants are asked whether they took the drug according to the protocol.

For peer review only

Should the participant be unwilling to reschedule their appointment, data collected up until that point will be carried forward to the analysis stages and they will be withdrawn from the investigation. If a participant is unable to return for their second visit, follow-up questionnaires will be obtained by post.

Criteria for withdrawal

Participation in this study is voluntary and participants are free to withdraw from the trial at any time, without giving any reason. However they must seek advice from the researchers before doing so as discontinuing duloxetine treatment suddenly may cause drug withdrawal effects. Involvement may be also discontinued by the investigators in case of any significant adverse effect of duloxetine/placebo treatment, or when subjects require major changes to their ongoing pain management, during the course of the study.

Adverse events and data safety monitoring

All observed or reported adverse events (AEs) are recorded by the investigators. No AEs are expected in relation to the MRI scanning. Potential AEs are anticipated to be only related to drug intake. All adverse events are recorded and monitored until resolution, stabilisation, or until it has been shown that the study intervention is not the cause. Any serious adverse event (SAE) is recorded and reported to the research ethics committee (REC) as part of the annual reports.

Handling and storage of data and documents

All study staff and investigators endeavour to protect the rights of the trial participants to privacy and informed consent, and will adhere to the Data Protection Act, 1998. Personal data

For peer review only

is handled confidentially. Imaging, sample and questionnaire data are anonymised by assigning a unique participant identification code, containing the abbreviation of the study's name followed by a sequence number. The link between study identification code and individual's personal details is kept on the consent form and MR screening form.

Computer held data is kept securely and password protected. All data is stored on a secure dedicated server. Access to data is given to the investigators and is restricted by user identifiers and passwords. Copies of the raw imaging data is also stored on CD/DVD in a locked room in the Radiological Sciences premises as back-up data. Either double data entry or checks of 40% of data entry are to be performed in order to promote data quality.

Direct access is permitted when required to all source documents and other study documentation e.g. signed consent forms, for the purpose of study monitoring and audit and other lawful regulatory inspection by the chief investigator, sponsor's designee and inspection by relevant regulatory authorities.

The investigators have full access to the dataset without limiting contractual obligations.

Monitoring trial conduct

Trial conduct is subject to systems audit of the Trial Master File for inclusion of essential documents, permissions to conduct the trial, trial delegation log, curriculum vitae of trial staff and training received, local document control procedures, consent procedures and recruitment logs, adherence to procedures defined in the protocol (e.g. inclusion / exclusion criteria, correct randomisation, timeliness of visits), adverse event recording and reporting, drug accountability, pharmacy records and equipment calibration logs.

The trial coordinator or a nominated designee of the sponsor shall carry out a site systems audit according to local requirements.

For peer review only

Ethics and dissemination

The study was approved by the East Midlands – Nottingham 2 research ethics committee (18/EM/0189) in August 2014 and is being carried out under the principles of the Declaration of Helsinki (64th, 2013) and Good Clinical Practice standards. Any modifications to the original protocol must undergo review by the research ethics committee involved. The design, documentation and data collection have been additionally discussed and shaped according to opinion of a focus group formed by representatives of patients and the public. The first participant was recruited in December 2014 and the last follow-up visit is expected in September 2016. Data analysis will start in October 2016. Results will be disseminated in peer-reviewed journals and at scientific meetings and conferences, where patient data will be presented anonymously.

Funding

This work is supported by Arthritis Research UK (Grant 18769) and the University of Nottingham. The investigators have full access to the final dataset without limiting contractual agreements.

Study sponsor: University of Nottingham

Sponsor's reference: 13124

Contact name: Angela Shone

Address: Room B50, King's Meadow Campus, University of Nottingham, Lenton Lane, Nottingham, NG7 2NR, UK

Telephone: 0115 8467906

Email: sponsor@nottingham.ac.uk

For peer review only

Contributors

DPA and DAW designed the study and DR, DPA, RDK, CRT and RPM developed the research protocol. DR leads the study, is responsible for data acquisition and will conduct image and statistical analyses. WC will undertake image and statistical analysis. CRT provides statistical consultation. DR, HB and WC undertake recruitment and data collection. DR and DPA wrote the manuscript, which was edited by CRT and RDK. All authors approved the final manuscript and authorship eligibility will follow guidelines of biomedical journals.

Competing interests

The authors have no potential conflicts of interest with respect to the research, authorship and publication of this article.

References

1. Cross M, Smith E, Hoy D, et al. The global burden of hip and knee osteoarthritis: estimates from the global burden of disease 2010 study. *Annals of the rheumatic diseases* 2014:annrheumdis-2013-204763.
2. McAlindon TE, Bannuru RR, Sullivan M, et al. OARSI guidelines for the non-surgical management of knee osteoarthritis. *Osteoarthritis and cartilage* 2014;22(3):363-88.
3. Beswick AD, Wylde V, Gooberman-Hill R, et al. What proportion of patients report long-term pain after total hip or knee replacement for osteoarthritis? A systematic review of prospective studies in unselected patients. *BMJ open* 2012;2(1):e000435.
4. Ohtori S, Orita S, Yamashita M, et al. Existence of a neuropathic pain component in patients with osteoarthritis of the knee. *Yonsei medical journal* 2012;53(4):801-05.
5. Sanders D, Krause K, O'Muircheartaigh J, et al. Pharmacologic Modulation of Hand Pain in Osteoarthritis: A Double-Blind Placebo-Controlled Functional Magnetic Resonance Imaging Study Using Naproxen. *Arthritis & Rheumatology* 2015;67(3):741-51.

For peer review only

6. Vachon-Presseau E, Tétreault P, Petre B, et al. Corticolimbic anatomical characteristics predetermine risk for chronic pain. *Brain* 2016:aww100.
7. Tanasescu R, Cottam WJ, Condon L, et al. Functional reorganisation in chronic pain and neural correlates of pain sensitisation: A coordinate based meta-analysis of 266 cutaneous pain fMRI studies. *Neuroscience & Biobehavioral Reviews* 2016;68:120-33.
8. Petzke F, Jensen K, Kosek E, et al. Using fMRI to evaluate the effects of milnacipran on central pain processing in patients with fibromyalgia. *Scandinavian Journal of Pain* 2013;4(2):65-74.
9. Giménez M, Pujol J, Ali Z, et al. Naproxen effects on brain response to painful pressure stimulation in patients with knee osteoarthritis: a double-blind, randomized, placebo-controlled, single-dose study. *The Journal of rheumatology* 2014;41(11):2240-48.
10. Duff EP, Vennart W, Wise RG, et al. Learning to identify CNS drug action and efficacy using multistudy fMRI data. *Science translational medicine* 2015;7(274):274ra16-74ra16.
11. Tracey I. Can neuroimaging studies identify pain endophenotypes in humans? *Nature Reviews Neurology* 2011;7(3):173-81.
12. Wager TD, Atlas LY, Lindquist MA, et al. An fMRI-based neurologic signature of physical pain. *New England Journal of Medicine* 2013;368(15):1388-97.
13. Brown GG, Perthen JE, Liu TT, et al. A primer on functional magnetic resonance imaging. *Neuropsychology review* 2007;17(2):107-25.
14. Petersen E, Zimine I, Ho YL, et al. Non-invasive measurement of perfusion: a critical review of arterial spin labelling techniques. *The British journal of radiology* 2014
15. Chappell AS, Ossanna MJ, Liu-Seifert H, et al. Duloxetine, a centrally acting analgesic, in the treatment of patients with osteoarthritis knee pain: a 13-week, randomized, placebo-controlled trial. *Pain* 2009;146(3):253-60.
16. Chappell AS, Desaiah D, Liu-Seifert H, et al. A double-blind, randomized, placebo-controlled study of the efficacy and safety of duloxetine for the treatment of chronic pain due to osteoarthritis of the knee. *Pain Practice* 2011;11(1):33-41.
17. Van Marle HJ, Tendolkar I, Urner M, et al. Subchronic duloxetine administration alters the extended amygdala circuitry in healthy individuals. *Neuroimage* 2011;55(2):825-31.
18. Fu CH, Costafreda SG, Sankar A, et al. Multimodal functional and structural neuroimaging investigation of major depressive disorder following treatment with duloxetine. *BMC psychiatry* 2015;15(1):1.
19. Freynhagen R, Baron R, Gockel U, et al. Pain DETECT: a new screening questionnaire to identify neuropathic components in patients with back pain. *Current medical research and opinion* 2006;22(10):1911-20.
20. Sullivan MJ, Bishop SR, Pivik J. The pain catastrophizing scale: development and validation. *Psychological assessment* 1995;7(4):524.
21. Spielberger CD. State-Trait anxiety inventory: Wiley Online Library 2010.
22. Beck AT, Steer RA, Brown GK. Beck depression inventory-II. *San Antonio, TX* 1996:78204-2498.

For peer review only

23. Hawker G, Davis A, French M, et al. Development and preliminary psychometric testing of a new OA pain measure–an OARSI/OMERACT initiative. *Osteoarthritis and Cartilage* 2008;16(4):409-14.
24. Ayearst LE, Harsanyi Z, Michalko KJ. The Pain and Sleep Questionnaire three-item index (PSQ-3): A reliable and valid measure of the impact of pain on sleep in chronic nonmalignant pain of various etiologies. *Pain Research and Management* 2012;17(4):281-90.
25. Cleeland C, Ryan K. Pain assessment: global use of the Brief Pain Inventory. *Annals of the Academy of Medicine, Singapore* 1994;23(2):129-38.
26. Borson S, Scanlan J, Brush M, et al. The Mini-Cog: a cognitive'vital signs' measure for dementia screening in multi-lingual elderly. *International journal of geriatric psychiatry* 2000;15(11):1021-27.
27. Pouli N, Nair RD, Lincoln NB, et al. The experience of living with knee osteoarthritis: exploring illness and treatment beliefs through thematic analysis. *Disability and rehabilitation* 2014
28. Jenkinson M, Beckmann CF, Behrens TE, et al. Fsl. *Neuroimage* 2012;62(2):782-90.
29. Hanke M, Halchenko YO, Sederberg PB, et al. PyMVPA: a unifying approach to the analysis of neuroscientific data. *Python in Neuroscience* 2015:157.
30. Song X-W, Dong Z-Y, Long X-Y, et al. REST: a toolkit for resting-state functional magnetic resonance imaging data processing. *PloS one* 2011;6(9):e25031.
31. Wang J, Wang X, Xia M, et al. GRETNA: a graph theoretical network analysis toolbox for imaging connectomics. *Frontiers in human neuroscience* 2015;9
32. Rubinov M, Sporns O. Complex network measures of brain connectivity: uses and interpretations. *Neuroimage* 2010;52(3):1059-69.
33. Provencher SW. Estimation of metabolite concentrations from localized in vivo proton NMR spectra. *Magnetic resonance in medicine* 1993;30(6):672-79.
34. Lenth RV. Some practical guidelines for effective sample size determination. *The American Statistician* 2001;55(3):187-93.
35. Lenth RV. Java applets for power and sample size [computer software]. *Retrieved October* 2006;31:2013.

For peer review only

Figure legends

Figure 1: Participation timeline and general characteristics of each study visit. QST, quantitative sensory testing; MRI, magnetic resonance imaging.

Figure 2: Details of experimental testing and randomisation of participants. Volume of interest for MRS is located in the mid anterior cingulate cortex. OA, osteoarthritis; QST, quantitative sensory testing; TS, temporal summation; PPT, pressure pain thresholds; CPM, conditioned pain modulation; Question, questionnaires; MRI, magnetic resonance imaging; fMRI, functional magnetic resonance imaging; rsfMRI, resting state fMRI; ASL, arterial spin labelling; T1w, T1-weighted MRI.

For peer review only

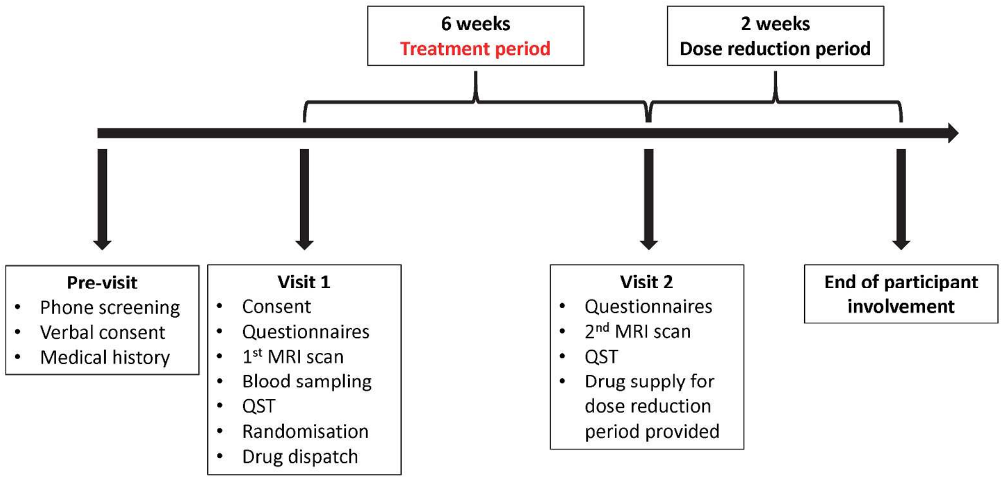

Participation timeline and general characteristics of each study visit. QST, quantitative sensory testing; MRI, magnetic resonance imaging

Figure 1

251x120mm (300 x 300 DPI)

For peer review only

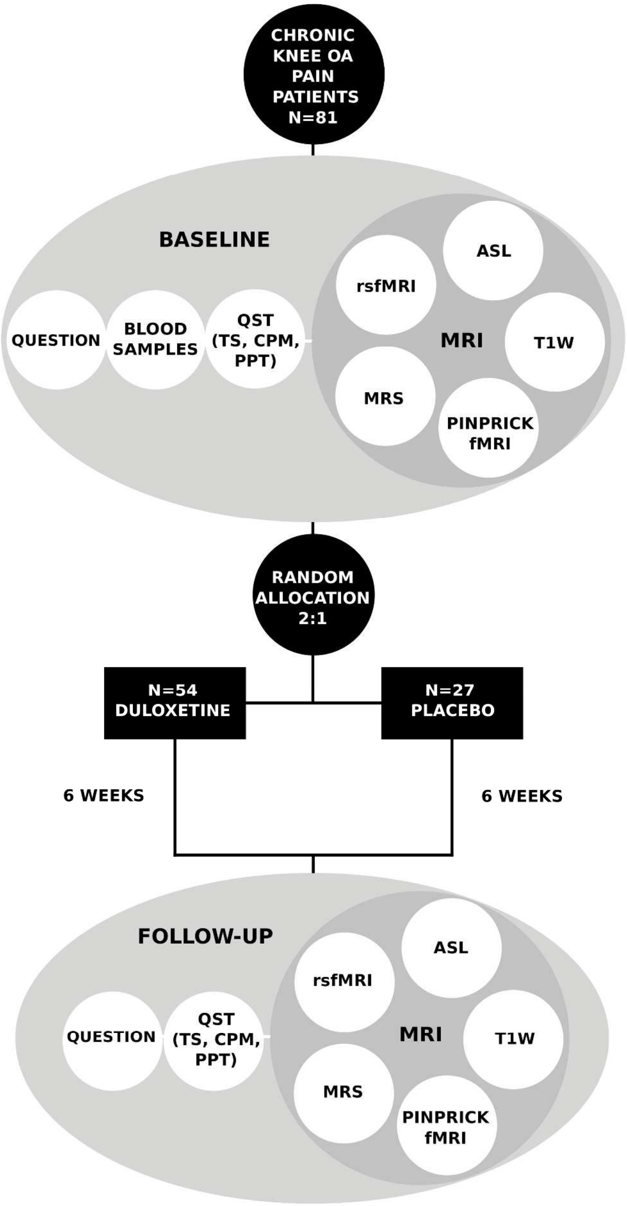

Details of experimental testing and randomisation of participants. Volume of interest for MRS is located in the mid anterior cingulate cortex. OA, osteoarthritis; QST, quantitative sensory testing; TS, temporal summation; PPT, pressure pain thresholds; CPM, conditioned pain modulation; Question, questionnaires; MRI, magnetic resonance imaging; fMRI, functional magnetic resonance imaging; rsfMRI, resting state fMRI; ASL, arterial spin labelling; T1w, T1-weighted MRI.

Figure 2

330x634mm (300 x 300 DPI)

SPIRIT

STANDARD PROTOCOL ITEMS: RECOMMENDATIONS FOR INTERVENTIONAL TRIALS

SPIRIT 2013 Checklist: Recommended items to address in a clinical trial protocol and related documents*

For peer review only

Section/item	Item No	Description	Addressed on page number
**Administrative information**			
Title	1	Descriptive title identifying the study design, population, interventions, and, if applicable, trial acronym	______1______
Trial registration	2a	Trial identifier and registry name. If not yet registered, name of intended registry	______2______
	2b	All items from the World Health Organization Trial Registration Data Set	______ok_____
Protocol version	3	Date and version identifier	______NA______
Funding	4	Sources and types of financial, material, and other support	______2______
Roles and responsibilities	5a	Names, affiliations, and roles of protocol contributors	______1,16___
	5b	Name and contact information for the trial sponsor	__18, 19______
	5c	Role of study sponsor and funders, if any, in study design; collection, management, analysis, and interpretation of data; writing of the report; and the decision to submit the report for publication, including whether they will have ultimate authority over any of these activities	_____10_______
	5d	Composition, roles, and responsibilities of the coordinating centre, steering committee, endpoint adjudication committee, data management team, and other individuals or groups overseeing the trial, if applicable (see Item 21a for data monitoring committee)	_____NA_____

For peer review only - http://bmjopen.bmj.com/site/about/guidelines.xhtml

Introduction

Background and rationale	6a	Description of research question and justification for undertaking the trial, including summary of relevant studies (published and unpublished) examining benefits and harms for each intervention	____3,4,5,6_____
	6b	Explanation for choice of comparators	___________
Objectives	7	Specific objectives or hypotheses	___5,6,7______
Trial design	8	Description of trial design including type of trial (eg, parallel group, crossover, factorial, single group), allocation ratio, and framework (eg, superiority, equivalence, noninferiority, exploratory)	___7,10_______

Methods: Participants, interventions, and outcomes

Study setting	9	Description of study settings (eg, community clinic, academic hospital) and list of countries where data will be collected. Reference to where list of study sites can be obtained	_____7_______
Eligibility criteria	10	Inclusion and exclusion criteria for participants. If applicable, eligibility criteria for study centres and individuals who will perform the interventions (eg, surgeons, psychotherapists)	_____7,8,9_____
Interventions	11a	Interventions for each group with sufficient detail to allow replication, including how and when they will be administered	_____10______
	11b	Criteria for discontinuing or modifying allocated interventions for a given trial participant (eg, drug dose change in response to harms, participant request, or improving/worsening disease)	_____15______
	11c	Strategies to improve adherence to intervention protocols, and any procedures for monitoring adherence (eg, drug tablet return, laboratory tests)	_____________
	11d	Relevant concomitant care and interventions that are permitted or prohibited during the trial	_____14______
Outcomes	12	Primary, secondary, and other outcomes, including the specific measurement variable (eg, systolic blood pressure), analysis metric (eg, change from baseline, final value, time to event), method of aggregation (eg, median, proportion), and time point for each outcome. Explanation of the clinical relevance of chosen efficacy and harm outcomes is strongly recommended	____12,13___
Participant timeline	13	Time schedule of enrolment, interventions (including any run-ins and washouts), assessments, and visits for participants. A schematic diagram is highly recommended (see Figure)	____9,10,11+Fig1

For peer review only - http://bmjopen.bmj.com/site/about/guidelines.xhtml

Sample size	14	Estimated number of participants needed to achieve study objectives and how it was determined, including clinical and statistical assumptions supporting any sample size calculations	______14______
Recruitment	15	Strategies for achieving adequate participant enrolment to reach target sample size	______14_____
**Methods: Assignment of interventions (for controlled trials)**			
Allocation:			
Sequence generation	16a	Method of generating the allocation sequence (eg, computer-generated random numbers), and list of any factors for stratification. To reduce predictability of a random sequence, details of any planned restriction (eg, blocking) should be provided in a separate document that is unavailable to those who enrol participants or assign interventions	______10_____
Allocation concealment mechanism	16b	Mechanism of implementing the allocation sequence (eg, central telephone; sequentially numbered, opaque, sealed envelopes), describing any steps to conceal the sequence until interventions are assigned	_____10______
Implementation	16c	Who will generate the allocation sequence, who will enrol participants, and who will assign participants to interventions	_____10______
Blinding (masking)	17a	Who will be blinded after assignment to interventions (eg, trial participants, care providers, outcome assessors, data analysts), and how	_____10______
	17b	If blinded, circumstances under which unblinding is permissible, and procedure for revealing a participant's allocated intervention during the trial	_____10______
**Methods: Data collection, management, and analysis**			
Data collection methods	18a	Plans for assessment and collection of outcome, baseline, and other trial data, including any related processes to promote data quality (eg, duplicate measurements, training of assessors) and a description of study instruments (eg, questionnaires, laboratory tests) along with their reliability and validity, if known. Reference to where data collection forms can be found, if not in the protocol	____10,11,12___
	18b	Plans to promote participant retention and complete follow-up, including list of any outcome data to be collected for participants who discontinue or deviate from intervention protocols	______15,16____

For peer review only - http://bmjopen.bmj.com/site/about/guidelines.xhtml

Data management	19	Plans for data entry, coding, security, and storage, including any related processes to promote data quality (eg, double data entry; range checks for data values). Reference to where details of data management procedures can be found, if not in the protocol	______17_______
Statistical methods	20a	Statistical methods for analysing primary and secondary outcomes. Reference to where other details of the statistical analysis plan can be found, if not in the protocol	_____13,14____
	20b	Methods for any additional analyses (eg, subgroup and adjusted analyses)	____13,14______
	20c	Definition of analysis population relating to protocol non-adherence (eg, as randomised analysis), and any statistical methods to handle missing data (eg, multiple imputation)	______13______
**Methods: Monitoring**			
Data monitoring	21a	Composition of data monitoring committee (DMC); summary of its role and reporting structure; statement of whether it is independent from the sponsor and competing interests; and reference to where further details about its charter can be found, if not in the protocol. Alternatively, an explanation of why a DMC is not needed	_____NA_______
	21b	Description of any interim analyses and stopping guidelines, including who will have access to these interim results and make the final decision to terminate the trial	_____NA______
Harms	22	Plans for collecting, assessing, reporting, and managing solicited and spontaneously reported adverse events and other unintended effects of trial interventions or trial conduct	_____15,16_____
Auditing	23	Frequency and procedures for auditing trial conduct, if any, and whether the process will be independent from investigators and the sponsor	______17_______
**Ethics and dissemination**			
Research ethics approval	24	Plans for seeking research ethics committee/institutional review board (REC/IRB) approval	_____2, 16____
Protocol amendments	25	Plans for communicating important protocol modifications (eg, changes to eligibility criteria, outcomes, analyses) to relevant parties (eg, investigators, REC/IRBs, trial participants, trial registries, journals, regulators)	_____16______

For peer review only - http://bmjopen.bmj.com/site/about/guidelines.xhtml

Consent or assent	26a	Who will obtain informed consent or assent from potential trial participants or authorised surrogates, and how (see Item 32)	____15_______
	26b	Additional consent provisions for collection and use of participant data and biological specimens in ancillary studies, if applicable	____14_________
Confidentiality	27	How personal information about potential and enrolled participants will be collected, shared, and maintained in order to protect confidentiality before, during, and after the trial	____16_______
Declaration of interests	28	Financial and other competing interests for principal investigators for the overall trial and each study site	____16,17_____
Access to data	29	Statement of who will have access to the final trial dataset, and disclosure of contractual agreements that limit such access for investigators	____17_______
Ancillary and post-trial care	30	Provisions, if any, for ancillary and post-trial care, and for compensation to those who suffer harm from trial participation	____ok_________
Dissemination policy	31a	Plans for investigators and sponsor to communicate trial results to participants, healthcare professionals, the public, and other relevant groups (eg, via publication, reporting in results databases, or other data sharing arrangements), including any publication restrictions	____18__________
	31b	Authorship eligibility guidelines and any intended use of professional writers	____16_______
	31c	Plans, if any, for granting public access to the full protocol, participant-level dataset, and statistical code	____NA_______
**Appendices**			
Informed consent materials	32	Model consent form and other related documentation given to participants and authorised surrogates	_____ok_______
Biological specimens	33	Plans for collection, laboratory evaluation, and storage of biological specimens for genetic or molecular analysis in the current trial and for future use in ancillary studies, if applicable	____ok_______

*It is strongly recommended that this checklist be read in conjunction with the SPIRIT 2013 Explanation & Elaboration for important clarification on the items. Amendments to the protocol should be tracked and dated. The SPIRIT checklist is copyrighted by the SPIRIT Group under the Creative Commons "Attribution-NonCommercial-NoDerivs 3.0 Unported" license.

For peer review only - http://bmjopen.bmj.com/site/about/guidelines.xhtml
